# The effects of gut microbiota colonizing on the porcine hypothalamus revealed by whole transcriptome analysis

**DOI:** 10.3389/fmicb.2022.970470

**Published:** 2022-10-13

**Authors:** Renli Qi, Jing Wang, Jing Sun, Xiaoyu Qiu, Xin Liu, Qi Wang, Feiyun Yang, Liangpeng Ge, Zuohua Liu

**Affiliations:** ^1^Chongqing Academy of Animal Science, Chongqing, China; ^2^Key Laboratory of Pig Industry Sciences, Ministry of Agriculture, Chongqing, China; ^3^College of Animal Science and Technology, Southwest University, Chongqing, China

**Keywords:** whole transcriptome, gene expression, germ-free pig, non-coding RNA genes, hypothalamus, microbiota-gut-brain axis

## Abstract

The roles of the microbe-gut-brain axis in metabolic homeostasis, development, and health are well-known. The hypothalamus integrates the higher nerve center system and functions to regulate energy balance, feeding, biological rhythms and mood. However, how the hypothalamus is affected by gut microbes in mammals is unclear. This study demonstrated differences in hypothalamic gene expression between the germ-free (GF) pigs and pigs colonized with gut microbiota (CG) by whole-transcriptome analysis. A total of 938 mRNAs, 385 lncRNAs and 42 miRNAs were identified to be differentially expressed between the two groups of pigs. An mRNA-miRNA-lncRNA competing endogenous RNA network was constructed, and miR-22-3p, miR-24-3p, miR-136-3p, miR-143-3p, and miR-545-3p located in the net hub. Gene function and pathway enrichment analysis showed the altered mRNAs were mainly related to developmental regulation, mitochondrial function, the nervous system, cell signaling and neurodegenerative diseases. Notably, the remarkable upregulation of multiple genes in oxidative phosphorylation enhanced the GF pigs’ hypothalamic energy expenditure. Additionally, the reduction in ATP content and the increase in carnitine palmitoyl transterase-1 (CPT1) protein level also confirmed this fact. Furthermore, the hypothalamic cell apoptosis rate in the CG piglets was significantly higher than that in the GF piglets. This may be due to the elevated concentrations of pro-inflammatory factors produced by gut bacteria. The obtained results collectively suggest that the colonization of gut microbes has a significant impact on hypothalamic function and health.

## Introduction

The bacterial community living in the intestine is closely related to the growth, development, metabolism, health and disease of the host (Buffie and Pamer, 2013; Bibbò et al., 2016; Zafar and Saier, 2021). The intestinal microbiota dominated by the bacteria in the *Firmicutes* and *Bacteroides* phyla not only helps their host digest nutrients that are difficult to decompose, such as dietary fiber, but can also produce various bacterial metabolites, such as short-chain fatty acids (SCFAs), biogenic amines, and amino acid derivatives (Han et al., 2021; Jia et al., 2021; Qi et al., 2021a,b). These metabolites are transported into the blood circulation or immune system and then reach the brain, liver, pancreas, muscle and other tissues to play different physiological regulatory roles. Of note, the reduction in microbiota diversity or the invasion by pathogenic bacteria not only destroys gut health directly but may also lead to immune disorders and the formation of various of metabolic diseases (Kim and Jazwinski, 2018; Wang et al., 2021).

Many studies have demonstrated complex and efficient information exchange and interaction in the microbiota-gut-brain axis (Kelly et al., 2016; Dinan and Cryan, 2017; Agirman et al., 2021; Zhao et al., 2021). Bacteria in the gut affect the brain and central nervous system (CNS), mainly by bacterial functional metabolites. In addition, the bacteria can stimulate and induce diverse peptide hormones secreted by the gastrointestinal tract, such as gastrin, ghrelin, and PYY, which participate in CNS signaling (Gribble and Reimann, 2019; Martchenko et al., 2020; Farhadipour and Depoortere, 2021). Accumulating studies have pointed out that the colonization and succession of the intestinal bacterial community affect brain development and signal delivery in the CNS in humans and animals, which affects the cognition, emotion and appetite of the host (Dinan and Cryan, 2017; Ceppa et al., 2019; Rutsch et al., 2020; Han et al., 2021). More recently, a study indicated that a bacterial structural component, muropeptides from the cell wall, can directly reach the host’s brain, especially the hypothalamus, to play a regulatory role in appetite and body temperature by activating the receptor protein NOD2 (Gabanyi et al., 2022). In addition, the dysbiosis of gut microbiota caused by aging or drugs has been shown to be related to many cerebral and neurological diseases such as autism, Parkinson’s disease, emotional and affective disorders, and bulimia (Quigley, 2017; Sun and Shen, 2018). Therefore, uncovering how the gut microbiota affects brain development and function has important implications for maintaining and improving our health.

Clearly, understanding how the gut microbiota affects brain gene expression is very helpful to gain insights into the interaction in the microbiota-gut-brain axis. Some previous studies based on germ-free (GF) mice have shown that the deletion and colonization of intestinal microbiota directly changed the gene expression and metabolic function of the cerebral cortex or hippocampus using transcriptome and metabolome analysis (Chen et al., 2017; Zhou et al., 2020). The hypothalamus is located in the center of the brain and is considered to be the intersection center of the endocrine system and nervous system. The hypothalamus has important functions in regulating body temperature, food intake, hormone secretion and biological rhythm. However, there is no report on the effect of intestinal microbes on the hypothalamic gene expression profile to date.

In this study, a GF pig model without any intestinal bacteria was used to study the effect of bacterial colonization on gene expression in the hypothalamus. We analyzed and compared the profiles of mRNA, lncRNA and miRNA expression in the hypothalamus of GF pigs and pigs that colonized the gut microbiota (CG pigs) by whole transcriptome analysis, which can synchronously analyze the expression of protein-coding and noncoding genes (Li et al., 2018; Gu et al., 2020; Ma et al., 2020). Our results reveal the significant effects on the porcine hypothalamic gene expression profile caused directly by the change in gut microbe introduction; thus, the findings are helpful for finding new targets for information interactions in the microbiota-gut-brain axis.

## Materials and methods

### Animal and sample collection

Eight newborn GF piglets were obtained *via* hysterectomy from a multiparous Chinese Bama sow (a common local Chinese small pig breed). Four of them were introduced to gut microorganisms through the fecal microbiota transplant (FMT) method for colonization (CG piglets). GF piglets and CG piglets were reared in positive-pressure sterile fiberglass isolators (Class Biologically Clean Ltd., WI, United States; three piglets per isolator) with a heated floor at 32°C–35°C before 25 days and 28°C–30°C during 26–42 days. They were fed to satiety 5–7 times a day with an autoclave-sterilized cow’s milk-based formula prepared from condensed milk (Co60 γ-irradiated sterile, including 21% protein) before 25 days of age and then fed sterile compound feed until the 42nd day. All the GF and CG piglets were euthanized under isoflurane anesthesia at 42 days of age. Their hypothalamus were collected and cryopreserved for subsequent analyses. The study was approved by Ethics Committee of the Chongqing Academy of Animal Science (No. 2020012B).

### FMT

FMT in piglets was performed as our described previously (Qi et al., 2021a,b). Five candidate adult donor mother pigs were used in the current study and consumed a regular diet without antibiotics and probiotics for 6 weeks prior to feces collection. Hog cholera virus, porcine parvovirus, porcine circovirus-2, porcine reproductive syndrome virus, respiratory syndrome virus, pseudorabies virus, foot and mouth disease virus, and mycoplasma hyopneumoniae were detected in the pigs. Finally, one pig without any pathogen was used as the trial donor for FMT.

FMT was performed five times on four GF infant pigs from day 3 to day 7. Briefly, 2 g of fresh stool was collected from the donor pig and placed in an anaerobic sampling tube. The stool was homogenized in 100 ml of cold saline water, filtered and then settled by gravity for 5 min. Resuspend the bacterial community using 20 ml sterile saline after centrifugation and washing 2 times. The bacteria in suspension were mean count 8 × 10^8^ CFU/ml. All the operations were carried out in an anaerobic workstation. The bacterial suspension was administered *via* gavage to each recipient piglet for 1 ml per day.

### Total RNA extraction, RNA library preparation, and sequencing

Total RNA from the hypothalamus was extracted using TRIzol (Invitrogen, United States) according to the manufacturer’s instructions. Three hypothalamus tissue samples (1/4 hypothalamus) from each group were used for RNA extraction. The RNA concentration and purity were checked by OD A260/A280 (>1.8) and A260/A230 (>1.6), and the yield and quality were assessed using an Agilent 2100 Bioanalyzer (Agilent Technologies, United States) and RNA 6000 Nano LabChip Kit (Agilent Technologies, United States). The RNA integrity number (RIN) of extracted RNA was >7.0.

The preparation of whole transcriptome libraries and deep sequencing were performed by Marjorbio Technology Corp., Ltd. (Shanghai, China). Whole transcriptome libraries were constructed using the Ribo-Zero Magnetic Gold Kit (Illumina, United States) and the NEBNext® Ultra™ RNA Library Prep Kit for Illumina according to the manufacturer’s instructions. The paired-end libraries were prepared by Illumina TruSeq_RNA Library Preparation Kit v2 (Illumina, USA) and were sequenced following the standard procedure on an Illumina HiSeq platform, and paired-end reads with 150 nucleotides were generated.

Raw sequencing reads were checked for potential sequencing issues and contaminants using FastQC. Adapter sequences, primers, number of fuzzy bases (Ns), and reads with quality scores below 30 were trimmed. Clean reads were aligned to the reference genome (Sus scrofa 11.1, http://asia.ensembl.org/Sus_scrofa/Info/Index) using the TopHat 2.1 program and the resulting alignment files were reconstructed with the Cufflinks 2.0 program.

### Small RNA library construction and sequencing

Approximately 1 μg of total RNA was used to prepare small RNA library according to protocol of NEBNext® Small RNA Library Prep Set for Illumina. Single-end sequencing (50 bp) was performed on an Illumina Hiseq 2500 following the vendor’s recommended protocol. Raw reads obtained by the sequencing were checked for potential sequencing issues and contaminants using FastQC. Reads with a length < 10 and > 34 nt were discarded. The clean reads were aligned against the miRNA precursor/mature miRNA in miRBase 20.0^1^ to identify known miRNAs. The unannotated sequences were mapped to the pig genome to analyze their expression and distribution in the genome, and then used to predict potential novel miRNA candidates by Mireap.^2^ The read counts of each known miRNA were then normalized to the total counts of sequence reads mapped to the miRBase version 20.0 database.

### Sequencing data analyses

HTseq was used to count the genes and calculate the transcripts per kilobase million reads (TPMs) to evaluate the gene expression level. The differentially expressed mRNAs, lncRNAs, and miRNAs in GF and CG pigs were screened using the edgeR R package in the R software (version 3.5.2). Statistical significance was defined as log_2_| (fold change) | ≥ 1 and *Padjust* < 0.05. Volcano plots and heatmaps of DERNAs were plotted using the ggplots and heatmap packages.

### Gene function and pathway enrichment analysis

Gene Ontology (GO) enrichment analysis, Kyoto Encyclopedia of Genes and Genomes (KEGG) pathway enrichment analysis and Reactome pathway enrichment analysis of DEGs, were performed using the OmicShare tools, a free online platform for data analysis.^3^ The GO enrichment analysis was performed by the enriched GO function, in which DEGs were divided into three groups: molecular function (MF), cellular component (CC), and biology process (BP) *Padjust* < 0.05 was the threshold for significantly enriched GO terms and KEGG pathways. A *q-*value < 0.05 was the threshold for significantly enriched Reactome pathways. The potential contribution of the whole altered gene expression in the hypothalamus was also explored *via* Gene Set Enrichment Analysis (GSEA) software (version 4.1.0), especially on the KEGG analysis.

### Transcription factors analysis and lncRNA target analysis

Transcription factors (TFs) can potentially affect the DEGs in the dataset, which can be identified by regulatory impact factor (RIF) metrics. To identify TFs in all DEGs, all 158 TFs in 11 families of Sus scrofa were obtained at AnimalTFDB 3.0.^4^ The target relationship between lncRNAs and TFs was analyzed using RNAplex software.^5^ For the cis-regulation analysis, we predicted the target coding genes within 10 kb upstream and downstream of lncRNA; for the trans-regulation analysis, the target genes were predicted based on the free energy that is needed to form secondary structures between lncRNAs and mRNA sequences.

### Construction of the mRNA-DE miRNA-lncRNA triple regulatory network

According to the hypothesis of the ceRNA network, all potential connections among the differentially expressed mRNAs, miRNAs, and lncRNAs were examined. Using DE miRNA as a bridge, we linked the DE mRNAs and DE lncRNAs by searching the miRNA complementary sequences, and a triple network among DE miRNAs and their targeted lncRNAs and mRNAs was constructed. The target genes of the DE miRNAs were predicted using the microT scoring method (paring score ≥ 0.8) *via* the DIANA platform, and lncRNAs regulated by the DE miRNAs were identified through alignment with lncBase Version 2.0. The Pearson correlation coefficient between lncRNAs and mRNAs was calculated and only the pairs with coefficients greater than 0.4 were involved in the triple network. Finally, a topological network of DE mRNAs-DE miRNAs-DE lncRNAs was visualized using Cytoscape Version 3.7.1.

### Protein–protein interaction network construction

The Search Tool for the Retrieval of Interacting Genes (STRING)^6^ is a database for searching interactions that can show the direct physical interaction and the indirect functional correlation between proteins. We used the STRING database to identify pairwise relationships among the 17 DE mRNAs related to oxidative phosphorylation, and then a protein–protein interaction (PPI) network was built.

### Real-time quantitative PCR

Real time quantitative PCR (RT-qPCR) was performed to verify the expression levels of genes DE by the method of RNA-Seq. The total RNA was reverse-transcribed to cDNA using a PrimeScript™ RT reagent Kit (TaKaRa, Japan). PCR was performed using a Q6 qRT-PCR system (ABI, United States) with SYBR Premix Ex Taq. II (TaKaRa). The *β-actin* gene was used as reference gene. All of the reactions were prepared while using three replicates and the expression levels of genes were expressed as fold change using the 2^−△△CT^ method. Primer sequences are showed in Supplementary Table S1.

### ATP content and ATPase activity detection

Four tissue samples from each group (1/4 hypothalamus) were used for ATP content detection and ATPase activity analysis by using an ATP Content Assay Kit (BC0305), and a Micro Na^+^K^+^-ATPase Assay Kit (BC0065) according to the manufacturer’s protocol (Solarbio, China). The tissue samples were incubated with extraction reagent and then homogenized by ultrasonic cracking on ice. After centrifugation for 10 min, the supernatant was collected for ATP content and ATPase activity determinations, respectively.

The working principle of the ATP Content Assay Kit is briefly described as follows. The Glucose and ATP are catalyzed by hexokinase to produce glucose 6-phosphate, which is further catalytically dehydrogenated to produce NADPH. The NADPH shows a characteristic absorption peak at 340 nm. The content of ATP is proportional to that of NADPH.

The working principle of the ATPase Assay Kit is briefly described as follows. Na^+^K^+^-ATPase decomposes the intracellular ATP to generate ADP and inorganic phosphorus, and so the ATPase activity is determined by measuring the amount of inorganic phosphorus.

### Cell apoptosis analysis

Four tissue samples each group (1/4 hypothalamus) were used for the cell apoptosis analysis. Apoptotic cells can be detected by terminal deoxynucleotidyl transferase (TdT)-mediated dUTP nick end labeling (TUNEL) assay referenced the methods of Brannelly et al. (2018). The hypothalamus tissue of pigs was assayed using a TUNEL Apoptosis Assay Kit (Solarbio) according to the manufacturer’s protocol. Frozen tissue sections of 5 μm thickness were incubated with H_2_O_2_ (0.3% H_2_O_2_ in methanol) at room temperature (RT) for 30 min. After rinsing with 0.01 mol/l PBS for three times, the sections were treated with TUNEL working solution (Component A) at 37°C for 60 min, and then rinsed twice with PBS. Add 100 ul reaction buffer (Component B) to each sections for 30s and remove it. Finally, the sections were observed under fluorescence microscope (DMi8, Leica, Germany).

### Western blotting assay

Three tissue samples (1/4 hypothalamus) were used for the WB detection. Total protein in the tissue was extracted by using an RIPA buffer (Beyotime, China). The standard western blotting method was used to determine the protein levels of CPT1, UCP2, total and cleaved of Caspase 3. GAPDH was used as a reference protein. Briefly, total protein extracts were separated by 12% SDS-PAGE and transferred to polyvinylidene membranes. The membranes were blocked with 5% nonfat milk in Tris-buffered saline containing 0.1% Tween 20 (TBST) at RT for 2 h, and probed overnight with primary antibodies at 4°C. After washing with TBST, the membranes were probed with the secondary antibody at RT for 2 h. The primary antibody for Caspase 3 was purchased from the Cell Signaling Technology (CST, #9664, United States). The primary antibodies for other protein were purchased from the Proteintech Group Co (UCP2, 11,081-1-AP; CPT1, 15,184-1-AP; GAPDH, 60004-1-Ig). Blots were visualized with a chemiluminescence reagent (Millipore, MA, USA) using an automatic imaging system (BioRad, CA, United States).

### Blood concentrations of ILS and IL-1 detection

The venous blood of pigs was left standing for 6 h and then centrifuged to separate the serum. The concentrations of ILS and IL-1 in the serum samples were evaluated using commercial swine enzyme linked immune sorbent assay (ELISA) kits (Mlbio Co. Ltd., Shanghai, China) following the manufacturer’s instructions.

### Statistical analysis

Data are presented as the mean ± standard error of the mean (SEM). GraphPad Prism 8.5 software was used for statistical analysis and graph creation. The differences between groups were determined by the non-parametric Mann–Whitney test. Differences were considered statistically significant at *p* < 0.05.

## Results

### Results of sequencing and characteristics of transcripts

We determined tissue morphology of the hypothalamus, and all the hypothalamic histomorphology was good and with no obvious lesions. There was no clear difference between the two groups of pigs (Supplementary Figure 1).

**Figure 1:**
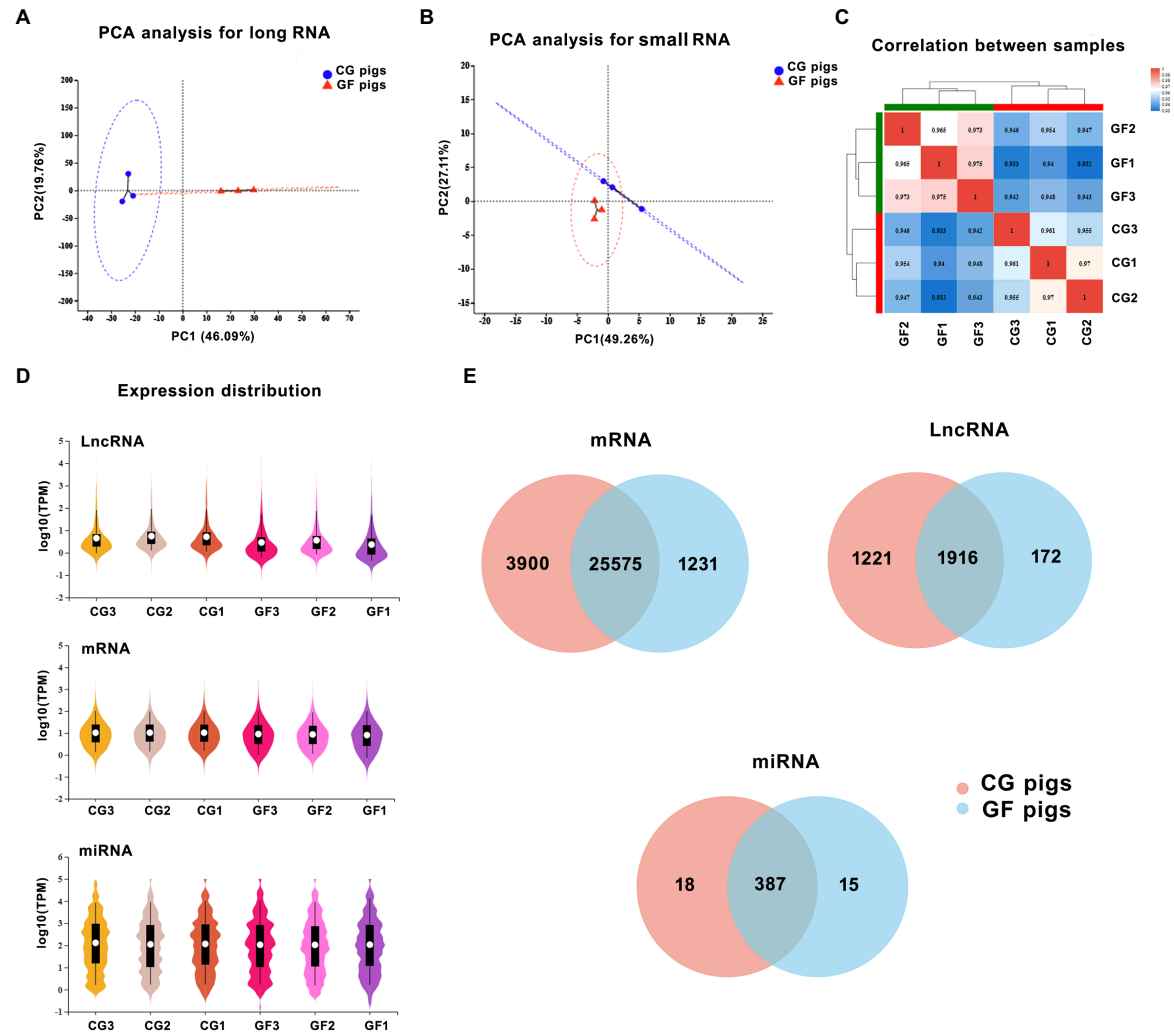
The features of the expression of long noncoding RNAs (lncRNAs), mRNAs, and microRNAs (miRNAs). **(A)** Principal component analysis (PCA) of two groups of samples based on the expression of long RNAs. **(B)** PCA of two groups of samples based on the expression of small RNAs. **(C)** The correlation analysis of samples. **(D)** The total expression levels of mRNAs, miRNAs, and lncRNAs between groups. **(E)** The number of identified lncRNAs, mRNAs, and miRNAs in the hypothalamus of the two groups of pigs.

From the whole-transcriptome sequencing, we identified a total of 50,167 transcripts for 19,256 known protein-coding genes (mRNAs), 14,393 known and 2,245 novel lncRNA transcripts, and 373 known and 539 novel miRNAs by sequencing (Figure 1).

Principal component analysis (PCA) showed that the gene expression profiles of the two groups of pigs, including long RNAs and small RNAs, were clearly distinguished (Figures 1A,B). In comparison, the two groups of pigs showed more differences in the expression profiles of long RNAs. Meanwhile, Spearman correlation analysis showed that the samples had high repeatability within the group (Figure 1C). This also reflected the good reproducibility of the sequencing results.

Expression levels of genes were indicated by the TPM values. By the comparison of the whole expression levels of mRNA, miRNA and lncRNA between groups, CG pigs had slightly higher expression levels of lncRNAs and mRNAs than GF pigs (Figure 1D). We found 25,575 (83.3%) mRNAs, 1,916 (57.9%) lncRNAs, and 387 (92.1%) miRNAs that were shared by both GF and CG pigs that showed in the Venn diagram in Figure 1E.

### Differentially expressed transcripts and genes

**Figure 2:**
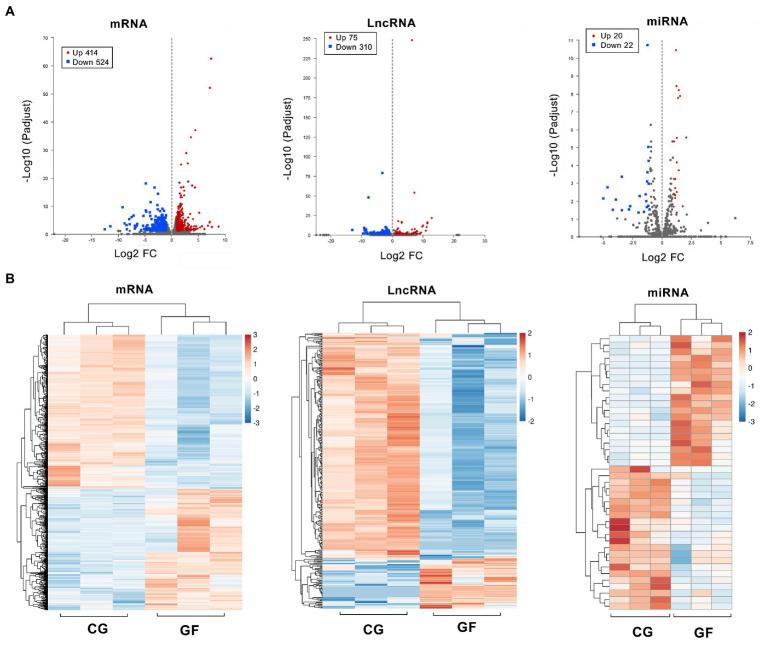
Differentially expressed transcripts and gene profiles in GF and CG pigs. **(A)** Volcano plots showing significantly different expression levels of different genes (mRNA) and transcripts (lncRNA and miRNA) between groups. **(B)** Clustering analysis of all differentially expressed genes and transcripts. The heatmap is based on expression values with log_2_| (fold change) | ≥ 1 and *Padjust* < 0.05.

The significant differences in transcript expression between GF and CG pigs are represented by a volcano plot (Figure 2A). There were 938 known mRNAs (including 414 upregulated and 524 downregulated protein-coding genes), 385 LncRNAs (including 75 upregulated and 315 downregulated transcripts) and 42 miRNAs (including 20 upregulated and 22 downregulated transcripts) that showed differential expression in the GF pigs relative to the CG pigs with log_2_| (fold change) | ≥ 1 and *Padjust* < 0.05. The heatmap in Figure 2B shows that the entire expression profiles of genes or transcripts in the two groups of pigs exhibited extremely significant differences. The top 10 upregulated and downregulated mRNAs, lncRNAs and miRNAs are shown in Supplementary Table S2. Obviously, the existence of a large number of differential expressed genes (DEGs) indicated the huge differences in the physiological status of the hypothalamus between the two groups of pigs.

### Gene ontology and Kyoto encyclopedia of genes and genomes analysis of DGEs

**Figure 3:**
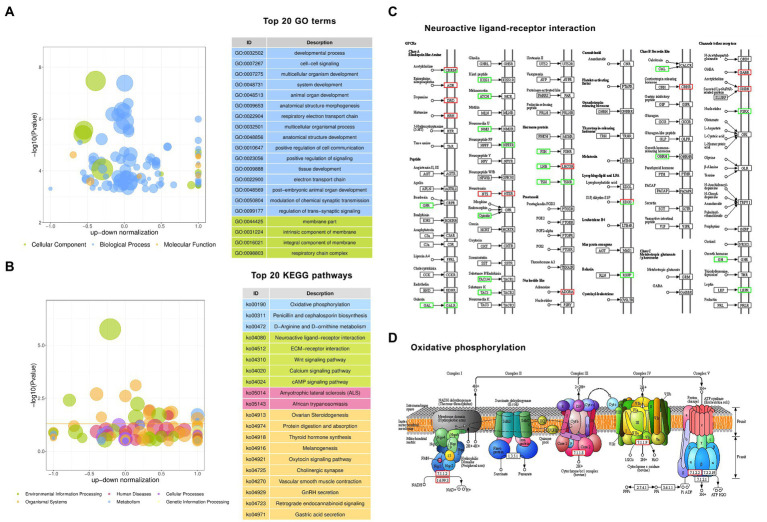
Gene Ontology (GO) and Kyoto Encyclopedia of Genes and Genomes (KEGG) enrichment analyses of differentially expressed protein-coding genes. **(A)** The top 20 enriched GO terms. **(B)** The top 20 enriched KEGG pathways. **(C)** The pathway of neuroactive ligand–receptor interaction. **(D)** The pathway of oxidative phosphorylation. Red boxes are upregulated genes, and green boxes are downregulated genes in GF pigs relative to CG pigs.

To better comprehend the mechanisms involved in hypothalamic development and function, gene ontology (GO) function and Kyoto encyclopedia of genes and genomes (KEGG) pathway enrichment analyses for the altered protein-coding genes were performed. According to the results of GO analysis, the DEGs were distributed across 136 significantly enriched functional terms with *Padjust* < 0.05, including 117 BP terms, 11 CC terms, and 8 MF terms. The top 20 significantly enriched terms are shown in Figure 3A. The five most significantly enriched GO terms were membrane part (CC, GO: 0044425, *Padjust* =  0.000), developmental process (BP, GO: 0032502, *Padjust* = 0.0002), cell–cell signaling (BP, GO: 0007267, *Padjust* = 0.0006), multicellular organism development (BP, GO: 0007275, *Padjust* = 0.0006) and system development (GO: 0048731, *Padjust* = 0.0006). The three most significantly enriched MF terms were hormone activity (MF, GO: 0005179, *Padjust* = 0.0080), NADH dehydrogenase (ubiquinone) activity (MF, GO: 0008137, *Padjust* = 0.0080), and NADH dehydrogenase (quinone) activity (MF, GO: 0050136, *Padjust* = 0.0006).

According to the results of KEGG analysis, the DEGs were significantly enriched in 21 pathways with *Padjust* < 0.05, which are shown in Figure 3B. The top 10 enriched pathways included neuroactive ligand–receptor interaction, ovarian steroidogenesis, protein digestion and absorption, thyroid hormone synthesis, ECM-receptor interaction, Wnt signaling pathway, amyotrophic lateral sclerosis (ALS), melanogenesis, oxytocin signaling pathway, cholinergic synapse, vascular smooth muscle contraction, and oxidative phosphorylation. Based on the analysis, we observed that there were 33 DEGs enriched in the neuroactive ligand–receptor interaction pathway, which was the 1st most significantly enriched pathway. Figure 3C shows the core composited factors and interactions in the pathway. This result suggests that the deletion of gut bacteria has a dramatic effect on hypothalamic neural signaling activated by multiple receptor proteins such as G protein-coupled receptors (GPCRs).

In addition, similar to the enriched MF terms in GO analysis, KEGG analysis also revealed dozens of DEGs related to the change in energy turnover by enrichment of the oxidative phosphorylation pathway. Figure 3D shows the core composited factors and interactions in the pathway.

### RT-qPCR confirmation of the DE genes

**Figure 4:**
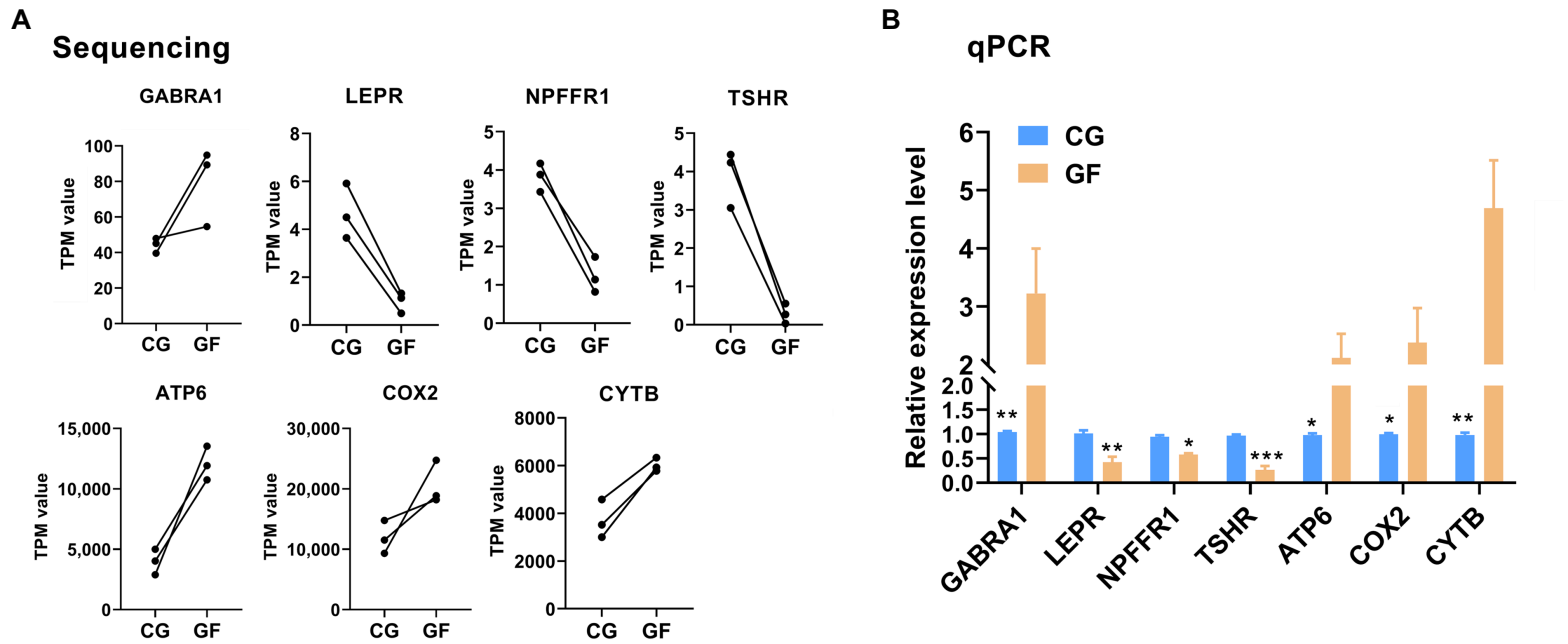
RT-qPCR validation of the RNA-Seq expression results. **(A)** The sequencing data for four DE genes (*NPFFR1*, *GABRA1*, *LEPR*, and *TSHR*) in the neuroactive ligand–receptor interaction and three DE genes (*COX2*, *ATP6*, and *CYTB*) in oxidative phosphorylation. **(B)** RT-qPCR data for the genes. The data are presented as the means ± SEM. *n* = 3. **indicates *p* < 0.01, * indicates *p* < 0.05 between CG pigs and GF pigs. *** indicates *p* < 0.001.

Next, to validate RNA-seq results, four DE genes (*NPFFR1*, *GABRA1*, *LEPR*, and *TSHR*) in the neuroactive ligand–receptor interaction and three DE genes (*COX2*, *ATP6*, and *CYTB*) in oxidative phosphorylation were selected for RT-qPCR assay. As shown in Figure 4, qPCR data for the expression of selected genes were coincided with the RNA-seq result, thereby indicating that our transcript identification and abundance estimation are highly reliable.

### Reactome pathway enrichment analysis of DEGs

**Figure 5:**
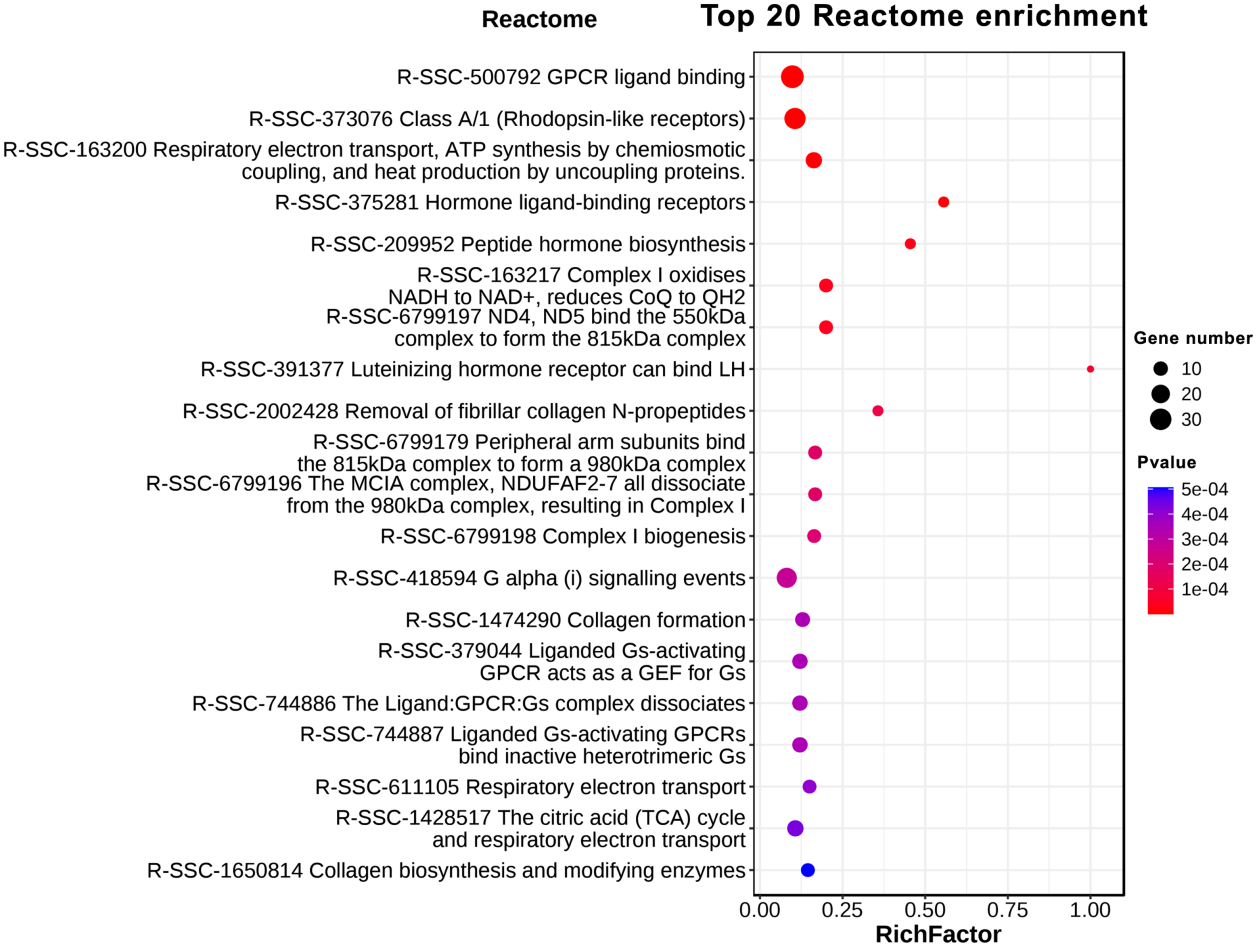
Reactome enrichment analysis of differentially expressed protein-coding genes.

Reactome is a knowledgebase of reactions and pathways for humans and dozens of other creatures, including pigs. Reactome provides an integrated view of the molecular details of biological processes ranging from metabolism to DNA replication and repair to signaling cascades (Fabregat et al., 2017; Jassal et al., 2020). Our Reactome analysis indicated that the DEGs were involved in 23 Reactome pathways with *q-*value < 0.05 (Figure 5). The Top 10 Reactome pathways were GPCR ligand binding (R-SSC-500792); Class A/1 (Rhodopsin-like receptors; R-SSC-373076); Respiratory electron transport, ATP synthesis by chemiosmotic coupling, and heat production by uncoupling proteins (R-SSC-163200); Hormone ligand-binding receptors (R-SSC-375281); Peptide hormone biosynthesis (R-SSC-209952); Complex I oxidizes NADH to NAD+, reduces CoQ to QH2 (R-SSC-6799197); ND4, ND5 bind the 550 kDa complex to form the 815 kDa complex (R-SSC-163217); Luteinizing hormone receptor can bind LH (R-SSC-391377); Removal of fibrillar collagen N-propeptides (R-SSC-2002428); Peripheral arm subunits bind the 815 kDa complex to form a 980 kDa complex (R-SSC-6799179). From the analysis of results, we found that GPCR protein family-mediated signaling, hormone signaling, heat production and mitochondrial oxidative metabolism showed clear differences in GF pigs compared to CG pigs.

### Transcription factor analysis

**Figure 6:**
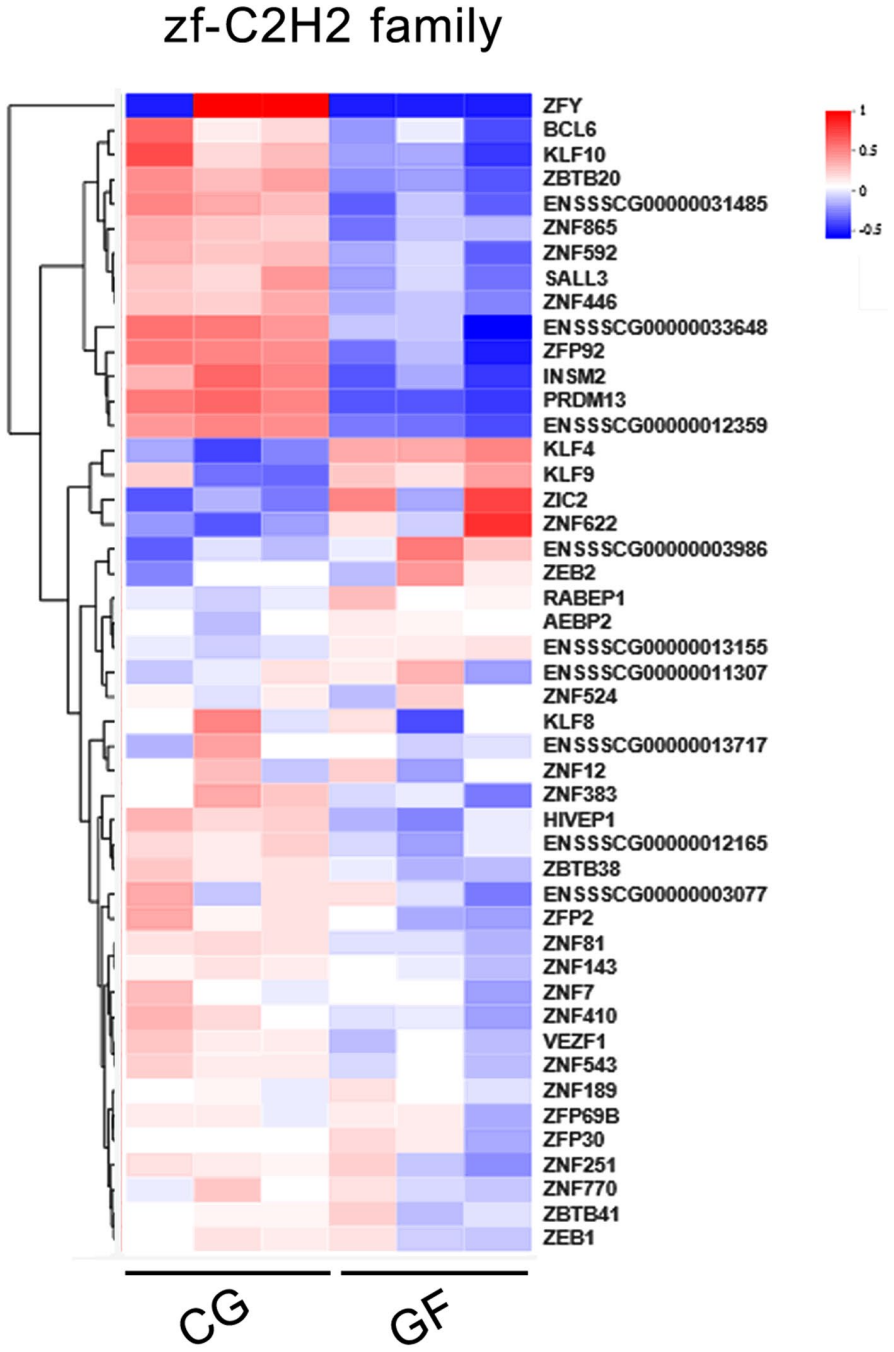
Clustering heatmap showing 47 altered *zf-C2H2* TFs.

In this study, the prediction of transcription factors (TFs) for the whole dataset of DEGs was performed, and 158 TFs were found among 938 DEGs, which showed differences in expression levels between CG and GF pigs. The differentially expressed TFs mainly included members of the *zf-C2H2* (47 TFs), *ZBTB* (15 TFs), *Homeobox* (11 TFs), *bHLH* (9 TFs), and *ARID* (5 TFs) gene families. The zf-C2H2 TF family has hundreds of members and is the largest superfamily in mammalian genomes, and these TFs are widely involved in the processes of cell proliferation, differentiation, death and intracellular signaling. The heatmap in Figure 6 shows the expression profiles of 47 TFs in the *zf-C2H2* family. More than 2/3 of the TFs in the *zf-C2H2* family showed significant reductions in expression levels in GF pigs, including multiple *ZNF* genes and *ZBTB* genes. These findings reveal the importance of gut microbiota for the normal expression and functions of *zf-C2H2* TFs. In addition, 170 lncRNAs have significant regulatory relationship with 6 of 47 *zf-C2H2* TFs according to the target prediction analysis shown in Supplementary Table S3. It has been observed that *INSM2* endured the most lncRNA regulation in the *zf-C2H2* family.

### Construction of a competing endogenous RNA regulatory network

**Figure 7:**
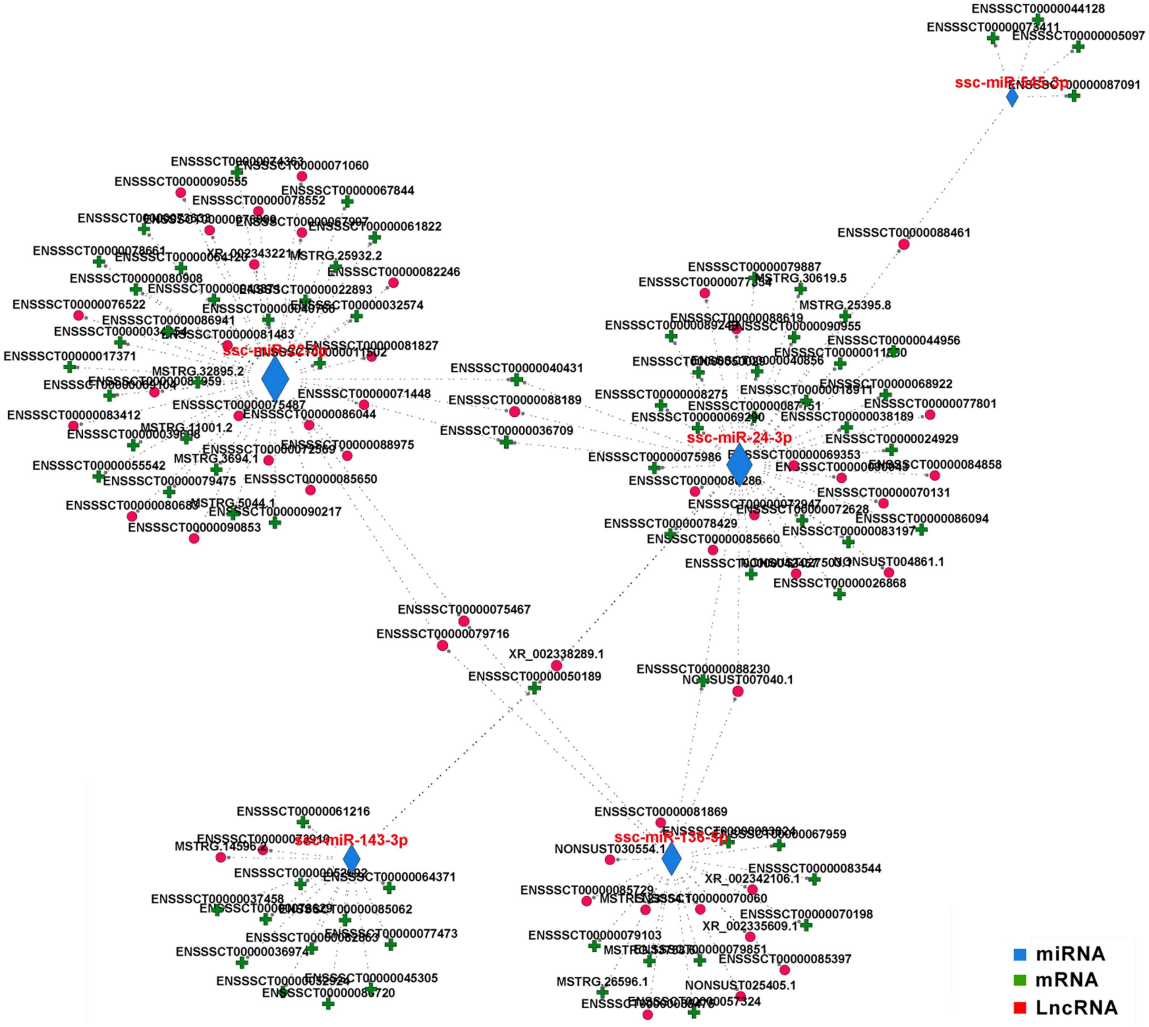
Competing endogenous (ce) RNA network in the hypothalamus. The ceRNA network was constructed based on lncRNA/miRNA and miRNA/mRNA interactions with correlation >0.9 and *padjust* < 0.05. The edges represent sequence matching, and lncRNAs connect expression-correlated mRNAs *via* miRNAs.

To investigate the interaction between different subtypes of DE RNAs, we constructed a competing endogenous RNA (ceRNA) network using DE miRNAs as a bridge for DE mRNAs and DE lncRNAs and identified the putative interactive pairs of DE miRNA – DE mRNA – DE lncRNA. 56 DE lncRNAs and 81 DE mRNAs were targeted by 5 DE miRNAs that were filtered according to *Padjust* < 0.05 and correlation > 0.90, and then these DE RNAs were used to construct an lncRNA-miRNA-mRNA triple network (Figure 7). In the regulatory ceRNA network, miR-22-3p, miR-24-3p, miR-136-3p, miR-143-3p, and miR-545-3p were located in the center and may play pivotal regulatory roles in the triple network. It is worth noting that all the five miRNAs were significantly downregulated in the hypothalamus of CG pigs. Target relationship between the five miRNAs and miRNAs or lncRNAs was showed in the Supplementary Table S4.

### Energic consumption was increased in GF pigs’ hypothalamus

**Figure 8:**
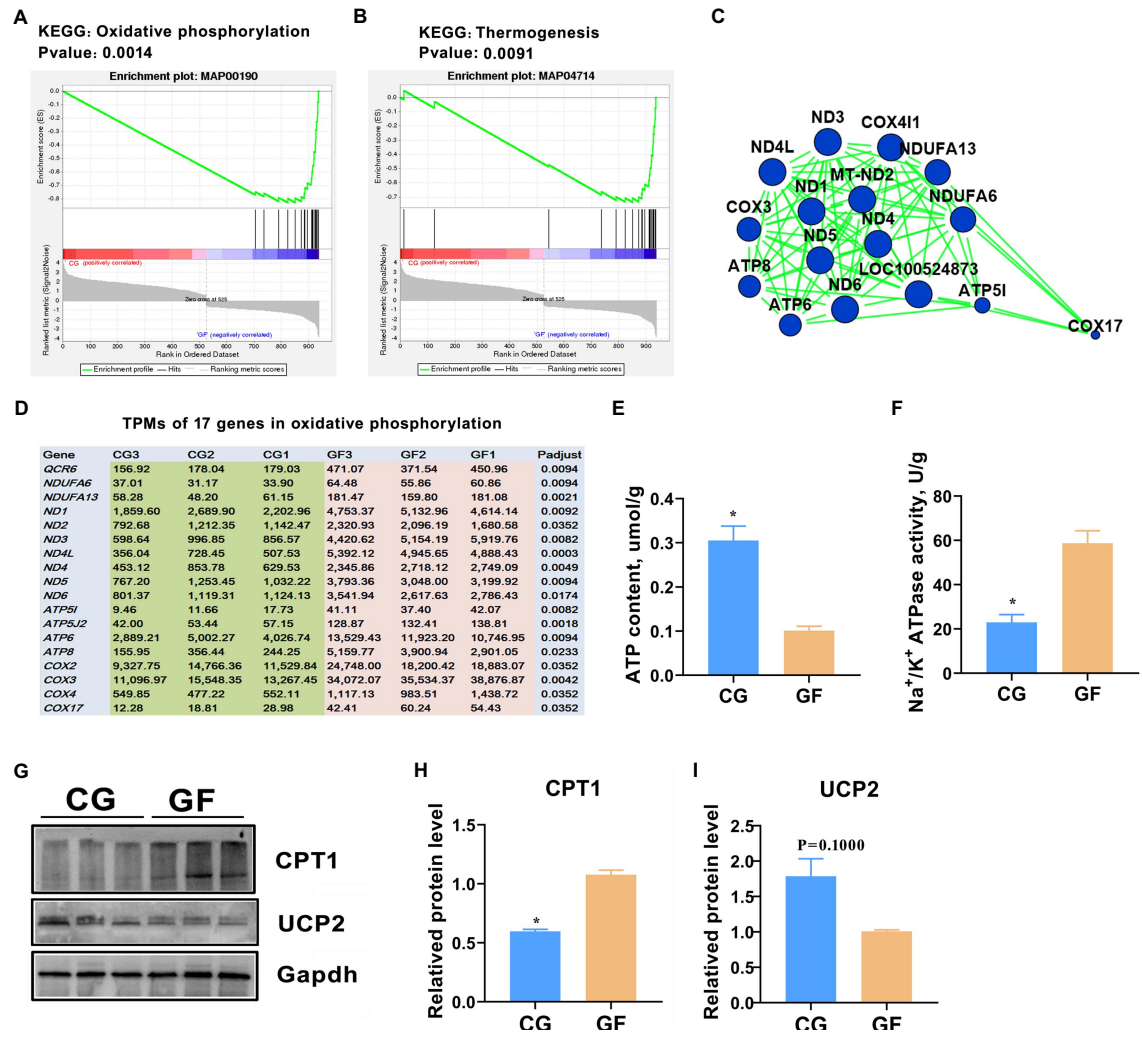
Hypothalamic energy consumption in pigs. GSEA analysis showed that oxidative phosphorylation **(A)** and thermogenesis **(B)** were significantly changed in GF pigs. **(C)** Protein–protein interaction (PPI) network of the 17 changed genes involved in oxidative phosphorylation and thermogenesis. **(D)** Expression levels (TPMs) of the 17 genes related to oxidative phosphorylation and thermogenesis. **(E)** ATP contents in the hypothalamus; **(F)** ATPase activity in the hypothalamus. **(G)** Western blotting (WB) analysis the protein levels of carnitine palmitoyl transterase-1 (CPT1) and uncoupling protein 2 (UCP2) in hypothalamus. **(H,I)** Quantitative analysis of the protein levels of CPT 1 and UCP2. The data are presented as the means ± SEM. *indicates *p* < 0.05 between CG pigs and GF pigs, *n* = 4 for the ATP content and ATPase activity assay and *n* = 3 for WB analysis.

Gene set enrichment analysis (GSEA) was also performed to identify the genes and cognitive pathways in the hypothalamus most affected by gut microbe colonization. Our GSEA results indicated that oxidative phosphorylation and thermogenesis were the most significantly enriched pathways (Figures 8A,B). This result was highly consistent with the KEGG and Reactome analyses. The two pathways share 17 altered genes. A protein–protein interaction (PPI) network was generated with the 17 genes and several mitochondrial DNA ND family genes located in the center of the network with the most neighbors and expanded nodes (Figure 8C). All of the genes were highly expressed in the GF pigs (Figure 8D), revealing that the requirement of energy expenditure was exorbitant.

To verify this result, we further evaluated the content of adenosine 5′-triphosphate (ATP), the principal energy currency of the cell, and the activity of ATPase in the hypothalamus. The obtained data showed that the ATP content in the GF pigs was significantly less than that in the CG pigs, with *p* < 0.05 (Figure 8E). However, the catalytic activity of Na^+^/K^+^ ATPase was almost 1.7-fold higher in the GF pigs than in the CG pigs (Figure 8F). Furthermore, protein level of carnitine palmitoyl transterase-1 (CPT-1) was clearly increased while protein level of uncoupling protein 2 (UCP2) was reduced in the GF pigs’ hypothalamus that was showed by the western blotting assay (Figures 8G–I). Previous studies indicated that CPT1 is a key rate-limiting enzyme in the process of mitochondrial fatty acid oxidation, while UCP2 has a role in the ATP production (Qu et al., 2016; Kim et al., 2019). Therefore, these findings indicating that ATP production was decreased, however, ATP consumption was enhanced in the pigs without gut microbes.

### Hypothalamic cell apoptosis was increased in the pigs with gut microbes

**Figure 9:**
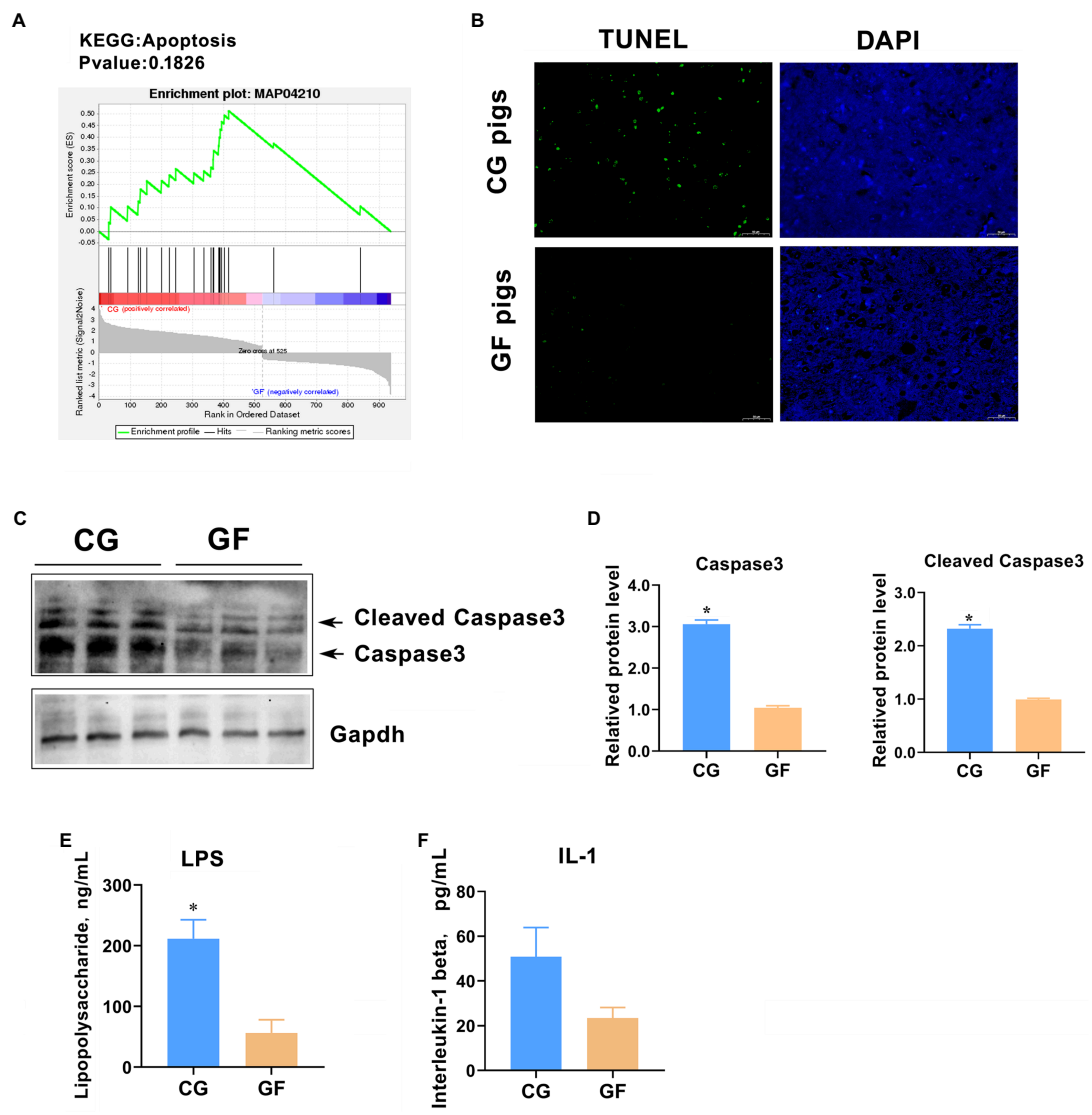
Hypothalamic cell apoptotic level between groups. **(A)** Apoptosis enrichment indicated by GSEA. **(B)** TUNEL analysis of cell apoptosis in the hypothalamus. Green color indicates TUNEL-positive cells. **(C)** Western blotting analysis of total and cleaved Caspase3. **(D)** Quantitative analysis of the protein levels of total and cleaved Caspase3. **(E)** Blood concentration of bacterial lipopolysaccharide (LPS). **(F)** Blood concentration of interleukin-1 (IL-1). The data are presented as the means ± SEM. *indicates *p* < 0.05 between CG pigs and GF pigs. *n* = 4 for the detection of LPS and IL-1 concentrations and *n* = 3 for WB analysis.

We noticed that the GSEA analysis also showed that dozens of DEGs were significantly enriched in apoptosis (Figure 9A). Therefore, we examined the hypothalamic cell apoptosis level in the two groups of pigs. The TdT-mediated dUTP nick end labeling (TUNEL) analysis showed that the number of apoptosis-positive cells in the hypothalamus of CG pigs was significantly greater than that of GF pigs (approximately 5–7-fold for GF pigs, Figure 9B). Furthermore, we analyzed the hypothalamic expression level of Caspase3, a key regulator and executor of programmed cell death (Jiang et al., 2020). The results of western blotting analysis showed that the protein levels of total caspase3 protein and cleaved Caspase3 (activated) were significantly increased in CG piglets with *p* < 0.05 (Figures 9C,D). It was speculated that the colonization of intestinal microbe increased the cell apoptosis rate in the hypothalamus in CG pigs may cause by the raise of circulating pro-inflammatory molecules. To confirm the speculation, blood concentrations of bacterial lipopolysaccharide (LPS) and interleukin-1 (IL-1) were detected by the ELISA method. The results showed that the blood concentration of LPS and IL-1 in CG pigs were about 5- and 2.3-fold of that of GF pigs, respectively (Figures 9E,F). Undoubtedly, these findings suggest that the colonization of gut microbes increases cell apoptosis in the hypothalamus, which may have a certain impact on brain health.

## Discussion

Accumulating evidence suggests that the colonization and proliferation of gut microbes closely affect and alter the brain and CNS in humans and animals (Kelly et al., 2016; Rutsch et al., 2020; Zafar and Saier, 2021). A healthy gut with diverse microbes is vital for normal brain functions and emotional behavior (Suganya and Koo, 2020). The brain is the most complex organ in our body, and it is indisputably difficult to clearly distinguish the subtle structural and physiological changes in the brain caused by gut microbiota. GF animals without gut microbes are ideal models for studying the microbe-gut-brain axis. Lu et al. (2018) investigated the differences in brain development and behaviors between GF mice and mice with a gut microbiota (Lu et al., 2018). They found that the mice with gut microbiota showed better brain structural maturation, better contextual memory, and better spatial and learning memory than GF mice. This suggests that commensal bacteria are necessary for normal morphological development and maturation of the brain.

To explore the molecules related to brain development and function, high-throughput omics analysis has been widely used, especially transcriptome analysis. Recently, several studies based on rodents have given explanatory notes about the effects of gut microbial deletion on the morphology, function, and gene expression profiles of the brain using transcriptome analysis. A previous study has showed that 50 miRNAs and 4,623 mRNAs were differentially expressed in the hippocampi of GF mice compared with those of specific pathogen-free (SPF) mice (Chen et al., 2017). Colonization with the gut bacterial community of SPF mice could partially correct the variation in mRNA and miRNA expression profiles in the hippocampus of GF mice, but that was not sufficient to reverse the behavioral alterations of GF mice. Similarly, Zhou investigated 2,230 differentially expressed lncRNAs in the hippocampus of GF mice relative to SPF mice. Most of these lncRNAs and their target mRNAs were highly associated with cardiac hypertrophy, nuclear factors of activated T cells (NFAT), gonadotropin-releasing hormone (GnRH), calcium, and cAMP-response element binding protein (CREB) signaling pathways (Zhou et al., 2020). These gene expression studies provide references for subsequent functional research on the microbiota-gut-brain axis.

Due to the similar organ size and similar metabolic characteristics of pigs and humans, pigs are considered to be a more suitable model for studying human-related metabolic diseases than mice and rats (Koopmans and Schuurman, 2015). In this study, the effects and details of the deletion and colonization of gut microbes on the pig hypothalamus, the core of the intersection of the nervous system and endocrine system, were demonstrated by whole transcriptome analysis; thus, this study provides a lot of valid information for future studies on brain development and metabolism. Our results showed that thousands of protein-coding RNAs and noncoding RNAs were clearly differentially expressed in GF pigs compared to pigs with gut microbes. These altered genes were involved in the regulation of the nervous system, signal transduction, developmental regulation, and neurological diseases, reflecting how gut microbes affect the health and function of the hypothalamus. Notably, the transcriptome analysis highlighted that ligand–receptor-mediated neural signaling systems, such as GPCR-mediated signaling networks, were significantly changed by gut microbe deletion. Undoubtedly, this leads to corresponding changes in CNS signaling and will affect the animal’s behavior and mood. In addition, we speculate that the changes in the ligand–receptor-activated neural signaling system in GF pigs were mainly due to the lack of bacterial metabolites such as short-chain fatty acids (SCFAs). As we all known that SCFAs could activate many members of GPCR protein family by receptor-ligand binding and then make an impact on activation of brain neural signals and keep of blood brain barrier (BBB; Silva et al., 2020). Moreover, some other types of compounds produced by gut bacteria, such as amino acid derivatives (i.e., melatonin and GABA) and secondary bile acids have similar effects on the switching in neural signaling.

Previous GF animal-based studies have indicated those energy homeostasis and glycolipid metabolisms are significantly altered in animals without any gut microbes (Krisko et al., 2020). A study showed that GF mice have a lower body fat weight and lower metabolic rate than mice with gut microbiota (Bäckhed et al., 2004). Nevertheless, Bäckhed found that compared to mice with a gut microbiota, GF mice are protected against the obesity that develops after consuming a high-fat, sugar-rich diet (Bäckhed et al., 2007). Mechanistically, GF mice have significantly increased levels of phosphorylated AMP-activated protein kinase (AMPK) and its downstream targets involved in fatty acid oxidation (acetyl-CoA carboxylase, ACC; carnitine palmitoyl transferase, CPT) in muscle and liver. A similar study reported that diet-induced obesity resistance in GF mice was conveyed by increased energy expenditure, preferential carbohydrate oxidation, and increased fecal fat and energy excretion (Kübeck et al., 2016). Our previous study also showed that GF piglets have smaller muscle and fat sizes than pigs with gut microbiota of the same age. Muscle cell mitochondria have dysregulated expression of multiple genes involved in oxidative metabolism (Qi et al., 2021a,b). The brain is the main organ for energy consumption in mammals, accounting for approximately 20% of the body’s energy expenditure (Bélanger et al., 2011; Engl and Attwell, 2015). This study found that the hypothalamus of GF and CG pigs showed large differences in oxidative phosphorylation and energy utilization. Gene expression analysis indicated that hypothalamic oxidative phosphorylation and mitochondrial function were significantly enhanced in GF pigs. Similarly, 31 genes related to oxidative phosphorylation and mitochondrial function was upregulated in the ilea of GF mice, as revealed by transcriptome analysis (Manes et al., 2017). These findings suggested that the gut microbiota could control bioenergetics for the host within the metabolic network by regulating various factors in energy turnover.

It is known that gut-residing microbes in the disease or disruption state may be involved in the progression of neurological disorders (Quigley, 2017; Sun and Shen, 2018). McVey-Neufeld et al. reported that colonization of gut microbiota restores normal intrinsic and extrinsic nerve function and gut–brain signaling in GF mice (McVey-Neufeld et al., 2015). Here, we unexpectedly found that colonization of the gut microbiota resulted in a significant increase in the number of apoptotic cells in the hypothalamus of piglets. This may be due to the increase in pro-inflammatory factors such as lipopolysaccharide (LPS) produced by gut microbes or by the imbalance of the cell repair process mediated by the immune system that was also affected by gut microorganisms. The significant elevation of blood concentrations of LPS and IL-1 in the CG pigs confirmed this speculation to a large extent. This study has not accurately explored and reflected the underlying molecular mechanism of cell apoptosis in the hypothalamus caused by gut microbiota, which is an important topic for our future research. This finding provides new evidence that the gut microbiota is inextricably linked to the health of the brain through crosstalk in the microbe-gut-brain axis.

We must state the limitations for this study. Limited by the experimental conditions, the sample size in this study is small, which may have a certain impact on the accuracy of results. In addition, the authenticity of the numerous changed genes and signaling targets obtained by the sequencing should to be verified through in-depth functional assays. We will continue to investigate the effects of gut microbes on hypothalamic function and health based on the GF pig model.

## Conclusion

To summarize, this study demonstrates the significant effects of gut microbe colonization on the porcine hypothalamus. The whole-transcriptome analysis showed thousands of protein coding and noncoding genes differentially expressed in GF pigs related to the pigs having a gut microbiota and therefore comprehensively affecting the development, function and health of the hypothalamus. Importantly, this study also provides new candidate target molecules for subsequent functional research.

## Data availability statement

The datasets presented in this study can be found in online repositories. The names of the repository/repositories and accession number(s) can be found at: NCBI GEO - GSE207675.

## Ethics statement

The study was approved by Ethics Committee of the Chongqing Academy of Animal Science (No. 2020012B).

## Author contributions

ZL and RQ: conceptualization. RQ, JW, XQ, XL, and JS: data curation. JS and XL: formal analysis. ZL, XQ, and RQ: funding acquisition. JW, QW, and XQ: investigation. JS, FY, and RQ: methodology. RQ and ZL: project administration. LG and RQ: resources. RQ: supervision and writing—original draft. XL and JW: visualization. LG and ZL: writing—review and editing. All authors contributed to the article and approved the submitted version.

## Funding

This research was supported by the National Natural Science Foundation of China (U21A20245) and the Financial Resourced Program of Chongqing (nos. 20238 and 21515).

## Conflict of interest

The authors declare that the research was conducted in the absence of any commercial or financial relationships that could be construed as a potential conflict of interest.

## Publisher’s note

All claims expressed in this article are solely those of the authors and do not necessarily represent those of their affiliated organizations, or those of the publisher, the editors and the reviewers. Any product that may be evaluated in this article, or claim that may be made by its manufacturer, is not guaranteed or endorsed by the publisher.

## Supplementary material

The Supplementary material for this article can be found online at: https://www.frontiersin.org/articles/10.3389/fmicb.2022.970470/full#supplementary-material

## Footnotes

^1^ http://www.mirbase.org/

^2^ http://sourceforge.net/projects/mireap/

^3^ https://www.omicshare.com/tools/

^4^ http://bioinfo.life.hust.edu.cn/AnimalTFDB/

^5^ http://www.tbi.univie.ac.at/software/

^6^ http://string-db.org
