# Supplementary material for: The effects of gut microbiota colonizing on the porcine hypothalamus revealed by whole transcriptome analysis
Source: Front Microbiol. 2022 Oct 13;13:970470. doi: 10.3389/fmicb.2022.970470 (PMC9606227; doi:10.3389/fmicb.2022.970470)
Supplement: Supplementary file 1 [file Table_1.DOCX]

**Supplementary table 1. Primer sequences of qPCR**

| **Gene** | **Primer sequence** (5’→3’) |
| --- | --- |
| NPFFR1 | F: CATCGTGCACCCTTTTCGTG  R: CGAGGGGCACATGATGAGAA |
| GABRA1 | F: ACCGCTCAGACCTAGTGAGT  R: CCAGGCCCAAAGGTAGTCAG |
| LEPR | F: GAGGCAGTTGTTGAAACGGA  R: TGTTCCAGTTTGCACCTGTT |
| TSHR | F: GCAGCTGGAATCACAGTCCT  R: GTTGCACAGGCCCTGAAAAG |
| COX2 | F: AAGACGCCACTTCACCCATC  R: TCTTGGGCATCCATTGTGCT |
| ATP6 | F: TGCCTCTTTCATTGCCCCTA  R: TTGGATCGAGATTGTGCGGT |
| CYTB | F: ACGCATTCATTGACCTCCCA  R: TGCTCCGTTTGCATGTAGGT |
| β-actin | F: GCGGCATCCACGAAACTAC  R: TGATCTCCTTCTGCATCCTGTC |
