# Supplementary material for: The effects of gut microbiota colonizing on the porcine hypothalamus revealed by whole transcriptome analysis
Source: Front Microbiol. 2022 Oct 13;13:970470. doi: 10.3389/fmicb.2022.970470 (PMC9606227; doi:10.3389/fmicb.2022.970470)
Supplement: Supplementary file 2 [file Table_2.DOCX]

**Supplementary table 2. top 10 upregulated and downregulated mRNAs, lncRNAs and miRNAs**

| **mRNA** | | | | | **lncRNA** | | | | **miRNA** | | | |
| --- | --- | --- | --- | --- | --- | --- | --- | --- | --- | --- | --- | --- |
| **Gene** | **name** | **Change** | **Log2（FC）** | ***Padjust*** | **Gene** | **Change** | **Log2（FC）** | ***Padjust*** | **Gene** | **Change** | **Log2（FC）** | ***Padjust*** |
| ENSSSCG00000026600 | KLHL30 | up | 8.88 | 0.0023 | ENSSSCT00000075948 | up | 12.86 | 0.0000 | miR-144 | up | 2.08 | 0.0000 |
| ENSSSCG00000025238 | SHOX2 | up | 7.57 | 0.0025 | ENSSSCT00000046229 | up | 11.45 | 0.0000 | miR-545-3p | up | 1.76 | 0.0202 |
| ENSSSCG00000007978 | —— | up | 7.42 | 0.0000 | NONSUST016307.1 | up | 11.41 | 0.0000 | miR-32 | up | 1.54 | 0.0000 |
| ENSSSCG00000014725 | HBB | up | 7.20 | 0.0000 | NONSUST013642.1 | up | 11.20 | 0.0000 | miR-490-5p | up | 1.43 | 0.0002 |
| ENSSSCG00000043911 | —— | up | 7.19 | 0.0000 | XR_002346056.1 | up | 10.95 | 0.0000 | miR-153 | up | 1.43 | 0.0000 |
| ENSSSCG00000046062 | —— | up | 7.16 | 0.0001 | NONSUST013638.1 | up | 10.00 | 0.0000 | miR-143-3p | up | 1.36 | 0.0000 |
| ENSSSCG00000038670 | —— | up | 6.83 | 0.0012 | ENSSSCT00000085926 | up | 9.80 | 0.0000 | miR-24-3p | up | 1.28 | 0.0000 |
| ENSSSCG00000003900 | DMBX1 | up | 6.14 | 0.0164 | NONSUST013655.1 | up | 9.40 | 0.0015 | miR-15a | up | 1.27 | 0.0001 |
| ENSSSCG00000014324 | MYOT | up | 6.14 | 0.0140 | XR_002341370.1 | up | 8.93 | 0.0131 | miR-146b | up | 1.27 | 0.0032 |
| ENSSSCG00000027275 | HHLA2 | up | 5.70 | 0.0015 | XR_002339514.1 | up | 8.16 | 0.0380 | miR-3613 | up | 1.26 | 0.0450 |
| ENSSSCG00000026430 | —— | down | -12.55 | 0.0187 | NONSUST013639.1 | down | -13.04 | 0.0000 | miR-375 | down | -5.00 | 0.0072 |
| ENSSSCG00000027046 | —— | down | -11.53 | 0.0018 | NONSUST013652.1 | down | -9.48 | 0.0068 | 17_41336 | down | -4.64 | 0.0017 |
| ENSSSCG00000042226 | —— | down | -9.20 | 0.0000 | ENSSSCT00000059733 | down | -9.39 | 0.0055 | 15_38177 | down | -4.19 | 0.0317 |
| ENSSSCG00000013328 | FSHB | down | -8.83 | 0.0001 | NONSUST023215.1 | down | -9.11 | 0.0000 | 2_7379 | down | -3.92 | 0.0082 |
| ENSSSCG00000012178 | —— | down | -8.49 | 0.0006 | NONSUST010494.1 | down | -9.06 | 0.0099 | ssc-miR-10386 | down | -3.42 | 0.0004 |
| ENSSSCG00000012179 | ZFY | down | -8.36 | 0.0212 | NONSUST023644.1 | down | -8.96 | 0.0000 | 2_7536 | down | -3.40 | 0.0326 |
| ENSSSCG00000032354 | KDM5D | down | -7.93 | 0.0000 | NONSUST020202.1 | down | -8.84 | 0.0000 | X_42979 | down | -2.81 | 0.0299 |
| ENSSSCG00000025434 | CGA | down | -7.85 | 0.0003 | NONSUST009878.1 | down | -8.70 | 0.0000 | miR-215 | down | -2.71 | 0.0202 |
| ENSSSCG00000015139 | BSX | down | -7.53 | 0.0000 | ENSSSCT00000069597 | down | -8.55 | 0.0000 | miR-224 | down | -2.00 | 0.0455 |
| ENSSSCG00000002775 | AGRP | down | -7.10 | 0.0000 | ENSSSCT00000067313 | down | -8.44 | 0.0001 | miR-205 | down | -1.90 | 0.0053 |
