# Supplementary material for: The effects of gut microbiota colonizing on the porcine hypothalamus revealed by whole transcriptome analysis
Source: Front Microbiol. 2022 Oct 13;13:970470. doi: 10.3389/fmicb.2022.970470 (PMC9606227; doi:10.3389/fmicb.2022.970470)
Supplement: Supplementary file 3 [file Table_3.DOCX]

**Supplementary table 3. DE LncRNA targeted zf-C2H2 TFs**

| **lncRNA gene id** | **Target gene** | **Regulate function** | ***Padjust*** |
| --- | --- | --- | --- |
| ENSSSCG00000042541 | INSM2 | trans | 0.000000 |
| ENSSSCG00000043353 | INSM2 | trans | 0.000000 |
| ENSSSCG00000045317 | PRDM13 | trans | 0.000314 |
| ENSSSCG00000048716 | INSM2 | trans | 0.000000 |
| ENSSSCG00000042466 | INSM2 | trans | 0.000000 |
| ENSSSCG00000047147 | ZNF622 | trans | 0.000000 |
| ENSSSCG00000049694 | KLF4 | trans | 0.000000 |
| ENSSSCG00000047147 | PRDM13 | trans | 0.000314 |
| ENSSSCG00000050913 | KLF4 | trans | 0.000000 |
| ENSSSCG00000036524 | INSM2 | trans | 0.000000 |
| ENSSSCG00000050369 | INSM2 | trans | 0.000000 |
| ENSSSCG00000047219 | INSM2 | trans | 0.000000 |
| ENSSSCG00000041282 | ZFP92 | trans | 0.000000 |
| ENSSSCG00000048330 | KLF4 | trans | 0.000000 |
| ENSSSCG00000042769 | KLF4 | trans | 0.000000 |
| ENSSSCG00000045043 | INSM2 | trans | 0.000000 |
| ENSSSCG00000045233 | INSM2 | trans | 0.000000 |
| ENSSSCG00000042955 | INSM2 | trans | 0.000000 |
| ENSSSCG00000050979 | INSM2 | trans | 0.000000 |
| ENSSSCG00000044412 | INSM2 | trans | 0.000000 |
| ENSSSCG00000051549 | INSM2 | trans | 0.000000 |
| ENSSSCG00000047138 | INSM2 | trans | 0.000000 |
| ENSSSCG00000050424 | INSM2 | trans | 0.000000 |
| ENSSSCG00000042799 | INSM2 | trans | 0.000000 |
| ENSSSCG00000050259 | INSM2 | trans | 0.000000 |
| ENSSSCG00000046194 | ZNF622 | trans | 0.000000 |
| ENSSSCG00000046194 | PRDM13 | trans | 0.000314 |
| ENSSSCG00000049833 | KLF4 | trans | 0.000000 |
| ENSSSCG00000048761 | INSM2 | trans | 0.000314 |
| ENSSSCG00000048096 | INSM2 | trans | 0.000000 |
| ENSSSCG00000050008 | PRDM13 | trans | 0.000314 |
| ENSSSCG00000050453 | INSM2 | trans | 0.000000 |
| ENSSSCG00000045392 | INSM2 | trans | 0.000000 |
| ENSSSCG00000038663 | INSM2 | trans | 0.000000 |
| ENSSSCG00000045536 | INSM2 | trans | 0.000000 |
| ENSSSCG00000041246 | INSM2 | trans | 0.000000 |
| ENSSSCG00000045402 | INSM2 | trans | 0.000000 |
| ENSSSCG00000049656 | INSM2 | trans | 0.000000 |
| ENSSSCG00000043775 | INSM2 | trans | 0.000000 |
| ENSSSCG00000051028 | ZFP92 | trans | 0.000000 |
| ENSSSCG00000050424 | INSM2 | trans | 0.000000 |
| ENSSSCG00000036880 | ZNF622 | trans | 0.000000 |
| ENSSSCG00000036880 | PRDM13 | trans | 0.000314 |
| ENSSSCG00000043386 | ZFP92 | trans | 0.000000 |
| ENSSSCG00000045101 | INSM2 | trans | 0.000000 |
| ENSSSCG00000042810 | INSM2 | trans | 0.000000 |
| ENSSSCG00000043231 | INSM2 | trans | 0.000000 |
| ENSSSCG00000050008 | PRDM13 | trans | 0.000314 |
| ENSSSCG00000045297 | INSM2 | trans | 0.000000 |
| ENSSSCG00000050582 | ZFP92 | trans | 0.000000 |
| ENSSSCG00000042712 | ZFP92 | trans | 0.000000 |
| ENSSSCG00000051274 | INSM2 | trans | 0.000000 |
| ENSSSCG00000047545 | ZNF622 | trans | 0.000000 |
| ENSSSCG00000047545 | PRDM13 | trans | 0.000314 |
| ENSSSCG00000046442 | INSM2 | trans | 0.000000 |
| ENSSSCG00000049497 | INSM2 | trans | 0.000000 |
| ENSSSCG00000050312 | INSM2 | trans | 0.000000 |
| ENSSSCG00000048190 | PRDM13 | trans | 0.000314 |
| ENSSSCG00000048820 | INSM2 | trans | 0.000000 |
| ENSSSCG00000048190 | ZNF622 | trans | 0.000000 |
| ENSSSCG00000047452 | INSM2 | trans | 0.000000 |
| ENSSSCG00000046986 | INSM2 | trans | 0.000000 |
| ENSSSCG00000046749 | INSM2 | trans | 0.000000 |
| ENSSSCG00000050149 | INSM2 | trans | 0.000000 |
| ENSSSCG00000048792 | INSM2 | trans | 0.000000 |
| ENSSSCG00000045021 | KLF4 | trans | 0.000000 |
| ENSSSCG00000041983 | PRDM13 | trans | 0.000314 |
| ENSSSCG00000049329 | INSM2 | trans | 0.000000 |
| ENSSSCG00000046050 | INSM2 | trans | 0.000000 |
| ENSSSCG00000041983 | ZNF622 | trans | 0.000000 |
| ENSSSCG00000043245 | PRDM13 | trans | 0.000314 |
| ENSSSCG00000043245 | ZNF622 | trans | 0.000000 |
| ENSSSCG00000050224 | INSM2 | trans | 0.000000 |
| ENSSSCG00000047004 | INSM2 | trans | 0.000000 |
| ENSSSCG00000049447 | INSM2 | trans | 0.000314 |
| ENSSSCG00000042133 | PRDM13 | trans | 0.000314 |
| ENSSSCG00000049257 | INSM2 | trans | 0.000000 |
| ENSSSCG00000047735 | PRDM13 | trans | 0.000314 |
| ENSSSCG00000047735 | ZNF622 | trans | 0.000000 |
| ENSSSCG00000042605 | INSM2 | trans | 0.000000 |
| ENSSSCG00000051505 | INSM2 | trans | 0.000000 |
| ENSSSCG00000046792 | KLF4 | trans | 0.000314 |
| ENSSSCG00000046792 | INSM2 | trans | 0.000314 |
| ENSSSCG00000043420 | INSM2 | trans | 0.000000 |
| ENSSSCG00000043966 | INSM2 | trans | 0.000000 |
| ENSSSCG00000046729 | INSM2 | trans | 0.000000 |
| ENSSSCG00000041752 | ZNF622 | trans | 0.000314 |
| ENSSSCG00000043503 | INSM2 | trans | 0.000000 |
| ENSSSCG00000047455 | ZNF622 | trans | 0.000000 |
| ENSSSCG00000047455 | PRDM13 | trans | 0.000314 |
| ENSSSCG00000050067 | ZNF622 | trans | 0.000000 |
| ENSSSCG00000050067 | PRDM13 | trans | 0.000314 |
| ENSSSCG00000035731 | KLF4 | trans | 0.000314 |
| ENSSSCG00000041752 | PRDM13 | trans | 0.002885 |
| ENSSSCG00000047616 | INSM2 | trans | 0.000000 |
| ENSSSCG00000046723 | KLF4 | trans | 0.000000 |
| ENSSSCG00000049100 | INSM2 | trans | 0.000000 |
| ENSSSCG00000043666 | INSM2 | trans | 0.000000 |
| ENSSSCG00000045086 | ZFP92 | trans | 0.000000 |
| ENSSSCG00000041907 | INSM2 | trans | 0.000000 |
| ENSSSCG00000048238 | INSM2 | trans | 0.000000 |
| ENSSSCG00000045127 | INSM2 | trans | 0.000000 |
| ENSSSCG00000033271 | INSM2 | trans | 0.000000 |
| ENSSSCG00000043604 | INSM2 | trans | 0.000000 |
| ENSSSCG00000042883 | INSM2 | trans | 0.000000 |
| ENSSSCG00000043063 | INSM2 | trans | 0.000000 |
| ENSSSCG00000046743 | ZNF622 | trans | 0.000000 |
| ENSSSCG00000046743 | PRDM13 | trans | 0.000314 |
| ENSSSCG00000051003 | PRDM13 | trans | 0.000314 |
| ENSSSCG00000051003 | ZNF622 | trans | 0.000000 |
| ENSSSCG00000041292 | INSM2 | trans | 0.000000 |
| ENSSSCG00000034990 | KLF4 | trans | 0.000000 |
| ENSSSCG00000043077 | INSM2 | trans | 0.000000 |
| ENSSSCG00000051731 | PRDM13 | trans | 0.000314 |
| ENSSSCG00000045778 | INSM2 | trans | 0.000000 |
| ENSSSCG00000051067 | INSM2 | trans | 0.000000 |
| ENSSSCG00000049064 | INSM2 | trans | 0.000000 |
| ENSSSCG00000041612 | ZFP92 | trans | 0.000000 |
| ENSSSCG00000045930 | INSM2 | trans | 0.000000 |
| ENSSSCG00000033103 | KLF4 | trans | 0.000000 |
| ENSSSCG00000002333 | PRDM13 | trans | 0.003010 |
| ENSSSCG00000040453 | KLF4 | trans | 0.000000 |
| ENSSSCG00000049737 | INSM2 | trans | 0.000000 |
| ENSSSCG00000050854 | INSM2 | trans | 0.000000 |
| ENSSSCG00000051318 | INSM2 | trans | 0.000000 |
| ENSSSCG00000044631 | INSM2 | trans | 0.000314 |
| ENSSSCG00000049091 | INSM2 | trans | 0.000000 |
| ENSSSCG00000042615 | ZNF622 | trans | 0.000000 |
| ENSSSCG00000009115 | INSM2 | trans | 0.000000 |
| ENSSSCG00000042615 | PRDM13 | trans | 0.000314 |
| ENSSSCG00000048753 | INSM2 | trans | 0.000000 |
| ENSSSCG00000044185 | INSM2 | trans | 0.000000 |
| ENSSSCG00000048948 | ZFP92 | trans | 0.000000 |
| ENSSSCG00000048891 | INSM2 | trans | 0.000000 |
| ENSSSCG00000049587 | INSM2 | trans | 0.001256 |
| ENSSSCG00000048754 | KLF4 | trans | 0.000314 |
| ENSSSCG00000048754 | INSM2 | trans | 0.000314 |
| ENSSSCG00000048776 | INSM2 | trans | 0.000000 |
| ENSSSCG00000050344 | KLF4 | trans | 0.000000 |
| ENSSSCG00000043662 | PRDM13 | trans | 0.000000 |
| ENSSSCG00000043662 | ZNF622 | trans | 0.000314 |
| ENSSSCG00000050971 | PRDM13 | trans | 0.000314 |
| ENSSSCG00000041031 | INSM2 | trans | 0.000000 |
| ENSSSCG00000032093 | INSM2 | trans | 0.000000 |
| ENSSSCG00000049768 | INSM2 | trans | 0.000000 |
| ENSSSCG00000043609 | INSM2 | trans | 0.000000 |
| ENSSSCG00000042119 | INSM2 | trans | 0.000000 |
| ENSSSCG00000051518 | PRDM13 | trans | 0.000314 |
| ENSSSCG00000050811 | ZFP92 | trans | 0.000000 |
| ENSSSCG00000041638 | KLF4 | trans | 0.000314 |
| ENSSSCG00000041979 | INSM2 | trans | 0.000000 |
| ENSSSCG00000048605 | INSM2 | trans | 0.000000 |
| ENSSSCG00000041475 | INSM2 | trans | 0.000000 |
| ENSSSCG00000050772 | PRDM13 | trans | 0.000314 |
| ENSSSCG00000046132 | PRDM13 | trans | 0.003010 |
| ENSSSCG00000050772 | ZNF622 | trans | 0.000000 |
| ENSSSCG00000046500 | INSM2 | trans | 0.000000 |
| ENSSSCG00000045731 | PRDM13 | trans | 0.000314 |
| ENSSSCG00000051518 | ZNF622 | trans | 0.000000 |
| ENSSSCG00000048856 | PRDM13 | trans | 0.002885 |
| ENSSSCG00000043061 | INSM2 | trans | 0.000314 |
| ENSSSCG00000048254 | ZFP92 | trans | 0.000000 |
| ENSSSCG00000045587 | INSM2 | trans | 0.000000 |
| ENSSSCG00000042143 | INSM2 | trans | 0.000000 |
| ENSSSCG00000043248 | KLF4 | trans | 0.000000 |
| ENSSSCG00000045640 | INSM2 | trans | 0.000000 |
| ENSSSCG00000032301 | KLF4 | trans | 0.000314 |
| ENSSSCG00000050008 | ZFP92 | trans | 0.000000 |
| ENSSSCG00000041905 | INSM2 | trans | 0.000000 |
| ENSSSCG00000048831 | INSM2 | trans | 0.000000 |
|  |  |  |  |
