# Supplementary material for: The effects of gut microbiota colonizing on the porcine hypothalamus revealed by whole transcriptome analysis
Source: Front Microbiol. 2022 Oct 13;13:970470. doi: 10.3389/fmicb.2022.970470 (PMC9606227; doi:10.3389/fmicb.2022.970470)
Supplement: Supplementary file 4 [file Table_4.DOCX]

**Supplementary table 4. Target relationships between miRNA/mRNA and miRNA/lncRNA**

| **miRNA** | **Target**  **transcript id** | **Type** | **Target**  **gene id** | **Correlation** | ***Padjust*** |
| --- | --- | --- | --- | --- | --- |
| miR-22-3p | ENSSSCT00000075487 | lncRNA | ENSSSCG00000050453 | -1.0000 | 0.0000 |
| miR-22-3p | ENSSSCT00000072569 | lncRNA | ENSSSCG00000038663 | -1.0000 | 0.0000 |
| miR-24-3p | ENSSSCT00000070131 | lncRNA | ENSSSCG00000045507 | -0.9429 | 0.0347 |
| miR-22-3p | ENSSSCT00000088189 | lncRNA | ENSSSCG00000050369 | -1.0000 | 0.0000 |
| miR-22-3p | ENSSSCT00000071060 | lncRNA | ENSSSCG00000050057 | -1.0000 | 0.0000 |
| miR-22-3p | ENSSSCT00000090555 | lncRNA | ENSSSCG00000049003 | -0.9429 | 0.0347 |
| miR-22-3p | ENSSSCT00000090853 | lncRNA | ENSSSCG00000050731 | -0.9429 | 0.0347 |
| miR-136-5p | ENSSSCT00000081869 | lncRNA | ENSSSCG00000046972 | -0.9429 | 0.0347 |
| miR-22-3p | ENSSSCT00000078552 | lncRNA | ENSSSCG00000051503 | -0.9856 | 0.0087 |
| miR-22-3p | ENSSSCT00000082246 | lncRNA | ENSSSCG00000048043 | -0.9429 | 0.0347 |
| miR-24-3p | ENSSSCT00000090943 | lncRNA | ENSSSCG00000044503 | -0.9429 | 0.0347 |
| miR-22-3p | ENSSSCT00000083412 | lncRNA | ENSSSCG00000051246 | -1.0000 | 0.0000 |
| miR-24-3p | NONSUST027503.1 | lncRNA | ENSSSCG00000050772 | -0.9429 | 0.0347 |
| miR-136-5p | ENSSSCT00000085475 | lncRNA | ENSSSCG00000049660 | -0.9429 | 0.0347 |
| miR-24-3p | ENSSSCT00000088189 | lncRNA | ENSSSCG00000050369 | -0.9429 | 0.0347 |
| miR-24-3p | ENSSSCT00000084858 | lncRNA | ENSSSCG00000049064 | -0.9429 | 0.0347 |
| miR-136-5p | ENSSSCT00000070060 | lncRNA | ENSSSCG00000045021 | -1.0000 | 0.0000 |
| miR-136-5p | ENSSSCT00000085397 | lncRNA | ENSSSCG00000047534 | -0.9429 | 0.0347 |
| miR-24-3p | NONSUST004861.1 | lncRNA | ENSSSCG00000046834 | -0.9276 | 0.0481 |
| miR-136-5p | XR_002335609.1 | lncRNA | ENSSSCG00000047709 | -1.0000 | 0.0000 |
| miR-22-3p | ENSSSCT00000079716 | lncRNA | ENSSSCG00000047138 | -1.0000 | 0.0000 |
| miR-24-3p | ENSSSCT00000077334 | lncRNA | ENSSSCG00000045233 | -0.9429 | 0.0347 |
| miR-24-3p | XR_002338289.1 | lncRNA | ENSSSCG00000042691 | -0.9429 | 0.0347 |
| miR-136-5p | ENSSSCT00000075467 | lncRNA | ENSSSCG00000043231 | -0.9429 | 0.0347 |
| miR-136-5p | ENSSSCT00000079716 | lncRNA | ENSSSCG00000047138 | -0.9429 | 0.0347 |
| miR-22-3p | ENSSSCT00000076099 | lncRNA | ENSSSCG00000045880 | -0.9429 | 0.0347 |
| miR-143-3p | XR_002338289.1 | lncRNA | ENSSSCG00000042691 | -0.9429 | 0.0347 |
| miR-22-3p | ENSSSCT00000086044 | lncRNA | ENSSSCG00000051440 | -0.9429 | 0.0347 |
| miR-22-3p | ENSSSCT00000081483 | lncRNA | ENSSSCG00000045402 | -1.0000 | 0.0000 |
| miR-22-3p | ENSSSCT00000075467 | lncRNA | ENSSSCG00000043231 | -1.0000 | 0.0000 |
| miR-22-3p | ENSSSCT00000076522 | lncRNA | ENSSSCG00000049458 | -1.0000 | 0.0000 |
| miR-22-3p | ENSSSCT00000080683 | lncRNA | ENSSSCG00000044023 | -0.9429 | 0.0347 |
| miR-24-3p | ENSSSCT00000077801 | lncRNA | ENSSSCG00000050051 | -0.9429 | 0.0347 |
| miR-22-3p | ENSSSCT00000088975 | lncRNA | ENSSSCG00000043857 | -0.9429 | 0.0347 |
| miR-136-5p | ENSSSCT00000085729 | lncRNA | ENSSSCG00000046070 | -0.9429 | 0.0347 |
| miR-545-3p | ENSSSCT00000088461 | lncRNA | ENSSSCG00000041959 | -0.9429 | 0.0347 |
| miR-24-3p | ENSSSCT00000085660 | lncRNA | ENSSSCG00000046432 | -0.9429 | 0.0347 |
| miR-24-3p | ENSSSCT00000088619 | lncRNA | ENSSSCG00000048831 | -0.9429 | 0.0347 |
| miR-136-5p | NONSUST030554.1 | lncRNA | ENSSSCG00000048298 | -0.9429 | 0.0347 |
| miR-136-5p | XR_002342106.1 | lncRNA | ENSSSCG00000043061 | -0.9276 | 0.0481 |
| miR-24-3p | ENSSSCT00000088461 | lncRNA | ENSSSCG00000041959 | -0.9429 | 0.0347 |
| miR-22-3p | ENSSSCT00000085650 | lncRNA | ENSSSCG00000046918 | -0.9429 | 0.0347 |
| miR-22-3p | ENSSSCT00000071448 | lncRNA | ENSSSCG00000043857 | -1.0000 | 0.0000 |
| miR-24-3p | ENSSSCT00000072947 | lncRNA | ENSSSCG00000041475 | -0.9429 | 0.0347 |
| miR-22-3p | ENSSSCT00000081827 | lncRNA | ENSSSCG00000050344 | -0.9429 | 0.0347 |
| miR-24-3p | NONSUST007040.1 | lncRNA | ENSSSCG00000043662 | -0.9276 | 0.0481 |
| miR-22-3p | ENSSSCT00000087959 | lncRNA | ENSSSCG00000051154 | -0.9429 | 0.0347 |
| miR-136-5p | NONSUST007040.1 | lncRNA | ENSSSCG00000043662 | -0.9276 | 0.0481 |
| miR-22-3p | XR_002343221.1 | lncRNA | ENSSSCG00000050887 | -0.9429 | 0.0347 |
| miR-136-5p | NONSUST025405.1 | lncRNA | ENSSSCG00000041979 | -0.9429 | 0.0347 |
| miR-22-3p | ENSSSCT00000067997 | lncRNA | ENSSSCG00000043317 | -0.9429 | 0.0347 |
| miR-24-3p | ENSSSCT00000086286 | lncRNA | ENSSSCG00000050157 | -0.9429 | 0.0347 |
| miR-143-3p | ENSSSCT00000073910 | lncRNA | ENSSSCG00000044369 | -0.9429 | 0.0347 |
| miR-24-3p | ENSSSCT00000069353 | lncRNA | ENSSSCG00000036846 | -0.9429 | 0.0347 |
| miR-136-5p | MSTRG.23354.1 | lncRNA | _ | -0.9429 | 0.0347 |
| miR-143-3p | MSTRG.14596.2 | lncRNA | _ | -1.0000 | 0.0000 |
| miR-22-3p | ENSSSCT00000040766 | mRNA | ENSSSCG00000011198 | -0.9429 | 0.0347 |
| miR-24-3p | ENSSSCT00000024929 | mRNA | ENSSSCG00000024563 | -0.9429 | 0.0347 |
| miR-24-3p | ENSSSCT00000018911 | mRNA | ENSSSCG00000017372 | -0.9429 | 0.0347 |
| miR-22-3p | ENSSSCT00000017371 | mRNA | ENSSSCG00000015954 | -1.0000 | 0.0000 |
| miR-143-3p | ENSSSCT00000052692 | mRNA | ENSSSCG00000013495 | -0.9429 | 0.0347 |
| miR-136-5p | ENSSSCT00000057324 | mRNA | ENSSSCG00000005225 | -0.9429 | 0.0347 |
| miR-24-3p | ENSSSCT00000083197 | mRNA | ENSSSCG00000029847 | -0.9429 | 0.0347 |
| miR-22-3p | ENSSSCT00000022893 | mRNA | ENSSSCG00000020671 | -1.0000 | 0.0000 |
| miR-545-3p | ENSSSCT00000044128 | mRNA | ENSSSCG00000002445 | -0.9411 | 0.0347 |
| miR-22-3p | ENSSSCT00000080908 | mRNA | ENSSSCG00000050792 | -1.0000 | 0.0000 |
| miR-24-3p | ENSSSCT00000078429 | mRNA | ENSSSCG00000043344 | -0.9429 | 0.0347 |
| miR-136-5p | ENSSSCT00000088230 | mRNA | ENSSSCG00000023693 | -0.9429 | 0.0347 |
| miR-136-5p | ENSSSCT00000079851 | mRNA | ENSSSCG00000048765 | -0.9429 | 0.0347 |
| miR-22-3p | ENSSSCT00000055542 | mRNA | ENSSSCG00000016075 | -0.9429 | 0.0347 |
| miR-22-3p | ENSSSCT00000069104 | mRNA | ENSSSCG00000049997 | -1.0000 | 0.0000 |
| miR-22-3p | ENSSSCT00000061822 | mRNA | ENSSSCG00000038478 | -0.9429 | 0.0347 |
| miR-22-3p | ENSSSCT00000039698 | mRNA | ENSSSCG00000013235 | -0.9429 | 0.0347 |
| miR-24-3p | ENSSSCT00000088230 | mRNA | ENSSSCG00000023693 | -0.9429 | 0.0347 |
| miR-136-5p | ENSSSCT00000083544 | mRNA | ENSSSCG00000050725 | -0.9429 | 0.0347 |
| miR-24-3p | ENSSSCT00000026868 | mRNA | ENSSSCG00000015175 | -0.9429 | 0.0347 |
| miR-143-3p | ENSSSCT00000082863 | mRNA | ENSSSCG00000044870 | -0.9429 | 0.0347 |
| miR-24-3p | ENSSSCT00000087151 | mRNA | ENSSSCG00000036210 | -0.9429 | 0.0347 |
| miR-136-5p | ENSSSCT00000083824 | mRNA | ENSSSCG00000045892 | -1.0000 | 0.0000 |
| miR-22-3p | ENSSSCT00000040431 | mRNA | ENSSSCG00000038825 | -1.0000 | 0.0000 |
| miR-24-3p | ENSSSCT00000038189 | mRNA | ENSSSCG00000031216 | -0.9429 | 0.0347 |
| miR-143-3p | ENSSSCT00000036974 | mRNA | ENSSSCG00000012699 | -0.9429 | 0.0347 |
| miR-22-3p | ENSSSCT00000011502 | mRNA | ENSSSCG00000010512 | -0.9429 | 0.0347 |
| miR-24-3p | ENSSSCT00000050029 | mRNA | ENSSSCG00000032010 | -0.9411 | 0.0347 |
| miR-143-3p | ENSSSCT00000086720 | mRNA | ENSSSCG00000028663 | -0.9429 | 0.0347 |
| miR-24-3p | ENSSSCT00000040856 | mRNA | ENSSSCG00000001918 | -0.9429 | 0.0347 |
| miR-143-3p | ENSSSCT00000050189 | mRNA | ENSSSCG00000012479 | -0.9429 | 0.0347 |
| miR-143-3p | ENSSSCT00000085062 | mRNA | ENSSSCG00000035000 | -0.9429 | 0.0347 |
| miR-545-3p | ENSSSCT00000087091 | mRNA | ENSSSCG00000044542 | -1.0000 | 0.0000 |
| miR-22-3p | ENSSSCT00000036709 | mRNA | ENSSSCG00000038825 | -1.0000 | 0.0000 |
| miR-22-3p | ENSSSCT00000064120 | mRNA | ENSSSCG00000015390 | -0.9429 | 0.0347 |
| miR-22-3p | ENSSSCT00000073633 | mRNA | ENSSSCG00000045829 | -1.0000 | 0.0000 |
| miR-24-3p | ENSSSCT00000068922 | mRNA | ENSSSCG00000011329 | -0.9429 | 0.0347 |
| miR-22-3p | ENSSSCT00000074363 | mRNA | ENSSSCG00000045121 | -1.0000 | 0.0000 |
| miR-24-3p | ENSSSCT00000086094 | mRNA | ENSSSCG00000048651 | -0.9429 | 0.0347 |
| miR-22-3p | ENSSSCT00000079475 | mRNA | ENSSSCG00000008263 | -0.9429 | 0.0347 |
| miR-545-3p | ENSSSCT00000073411 | mRNA | ENSSSCG00000008789 | -0.9276 | 0.0481 |
| miR-24-3p | ENSSSCT00000044956 | mRNA | ENSSSCG00000002640 | -0.9429 | 0.0347 |
| miR-22-3p | ENSSSCT00000032574 | mRNA | ENSSSCG00000003584 | -0.9429 | 0.0347 |
| miR-24-3p | ENSSSCT00000090955 | mRNA | ENSSSCG00000004123 | -0.9429 | 0.0347 |
| miR-24-3p | ENSSSCT00000011850 | mRNA | ENSSSCG00000010832 | -0.9429 | 0.0347 |
| miR-22-3p | ENSSSCT00000043871 | mRNA | ENSSSCG00000001611 | -0.9429 | 0.0347 |
| miR-143-3p | ENSSSCT00000052924 | mRNA | ENSSSCG00000037504 | -0.9429 | 0.0347 |
| miR-143-3p | ENSSSCT00000037458 | mRNA | ENSSSCG00000001641 | -0.9429 | 0.0347 |
| miR-143-3p | ENSSSCT00000076629 | mRNA | ENSSSCG00000041174 | -0.9429 | 0.0347 |
| miR-143-3p | ENSSSCT00000061216 | mRNA | ENSSSCG00000015273 | -0.9429 | 0.0347 |
| miR-22-3p | ENSSSCT00000078661 | mRNA | ENSSSCG00000049525 | -1.0000 | 0.0000 |
| miR-24-3p | ENSSSCT00000079887 | mRNA | ENSSSCG00000016082 | -0.9429 | 0.0347 |
| miR-24-3p | ENSSSCT00000008275 | mRNA | ENSSSCG00000007543 | -0.9429 | 0.0347 |
| miR-22-3p | ENSSSCT00000090217 | mRNA | ENSSSCG00000038144 | -1.0000 | 0.0000 |
| miR-545-3p | ENSSSCT00000005097 | mRNA | ENSSSCG00000004616 | -0.9429 | 0.0347 |
| miR-143-3p | ENSSSCT00000077473 | mRNA | ENSSSCG00000040157 | -0.9429 | 0.0347 |
| miR-24-3p | ENSSSCT00000075986 | mRNA | ENSSSCG00000009951 | -0.9429 | 0.0347 |
| miR-22-3p | ENSSSCT00000067844 | mRNA | ENSSSCG00000035873 | -0.9429 | 0.0347 |
| miR-24-3p | ENSSSCT00000050189 | mRNA | ENSSSCG00000012479 | -0.9429 | 0.0347 |
| miR-136-5p | ENSSSCT00000079103 | mRNA | ENSSSCG00000041425 | -0.9429 | 0.0347 |
| miR-24-3p | ENSSSCT00000042457 | mRNA | ENSSSCG00000039395 | -0.9429 | 0.0347 |
| miR-24-3p | ENSSSCT00000040431 | mRNA | ENSSSCG00000038825 | -0.9429 | 0.0347 |
| miR-143-3p | ENSSSCT00000064371 | mRNA | ENSSSCG00000003438 | -0.9411 | 0.0347 |
| miR-24-3p | ENSSSCT00000036709 | mRNA | ENSSSCG00000038825 | -0.9429 | 0.0347 |
| miR-136-5p | ENSSSCT00000070198 | mRNA | ENSSSCG00000039616 | -1.0000 | 0.0000 |
| miR-143-3p | ENSSSCT00000045305 | mRNA | ENSSSCG00000029875 | -1.0000 | 0.0000 |
| miR-136-5p | ENSSSCT00000067959 | mRNA | ENSSSCG00000010337 | -0.9429 | 0.0347 |
| miR-24-3p | ENSSSCT00000072628 | mRNA | ENSSSCG00000045235 | -0.9429 | 0.0347 |
| miR-22-3p | ENSSSCT00000086941 | mRNA | ENSSSCG00000005480 | -0.9429 | 0.0347 |
| miR-24-3p | ENSSSCT00000069290 | mRNA | ENSSSCG00000011714 | -0.9429 | 0.0347 |
| miR-24-3p | ENSSSCT00000089249 | mRNA | ENSSSCG00000004082 | -0.9429 | 0.0347 |
| miR-22-3p | ENSSSCT00000034854 | mRNA | ENSSSCG00000012403 | -0.9429 | 0.0347 |
| miR-22-3p | MSTRG.11001.2 | mRNA | ENSSSCG00000009779 | -1.0000 | 0.0000 |
| miR-136-5p | MSTRG.26596.1 | mRNA | ENSSSCG00000000179 | -0.9429 | 0.0347 |
| miR-24-3p | MSTRG.30619.5 | mRNA | ENSSSCG00000003531 | -0.9429 | 0.0347 |
| miR-22-3p | MSTRG.25932.2 | mRNA | ENSSSCG00000006887 | -0.9429 | 0.0347 |
| miR-22-3p | MSTRG.5044.1 | mRNA | ENSSSCG00000038251 | -1.0000 | 0.0000 |
| miR-22-3p | MSTRG.32895.2 | mRNA | ENSSSCG00000001538 | -1.0000 | 0.0000 |
| miR-136-5p | MSTRG.13758.6 | mRNA | ENSSSCG00000015883 | -1.0000 | 0.0000 |
| miR-24-3p | MSTRG.25395.8 | mRNA | ENSSSCG00000006626 | -0.9429 | 0.0347 |
| miR-22-3p | MSTRG.3694.1 | mRNA | ENSSSCG00000042888 | -1.0000 | 0.0000 |
