# Supplementary figures and images for: The effects of gut microbiota colonizing on the porcine hypothalamus revealed by whole transcriptome analysis
Source: Front Microbiol. 2022 Oct 13;13:970470. doi: 10.3389/fmicb.2022.970470 (PMC9606227; doi:10.3389/fmicb.2022.970470)

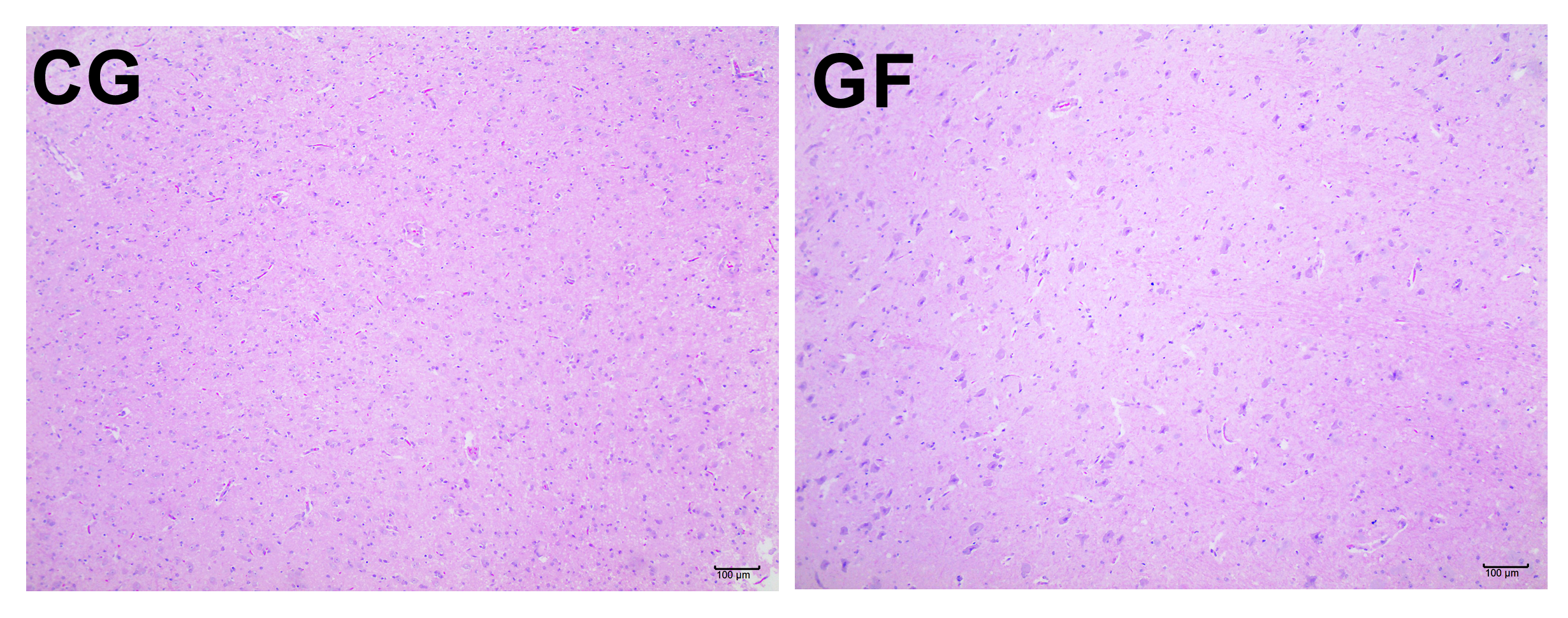

Supplement: Supplementary file 5 [file Image_1.TIF]
